# Supplementary figures and images for: Brain high-throughput multi-omics data reveal molecular heterogeneity in Alzheimer’s disease
Source: PLoS Biol. 2024 Apr 30;22(4):e3002607. doi: 10.1371/journal.pbio.3002607 (PMC11086901; doi:10.1371/journal.pbio.3002607)

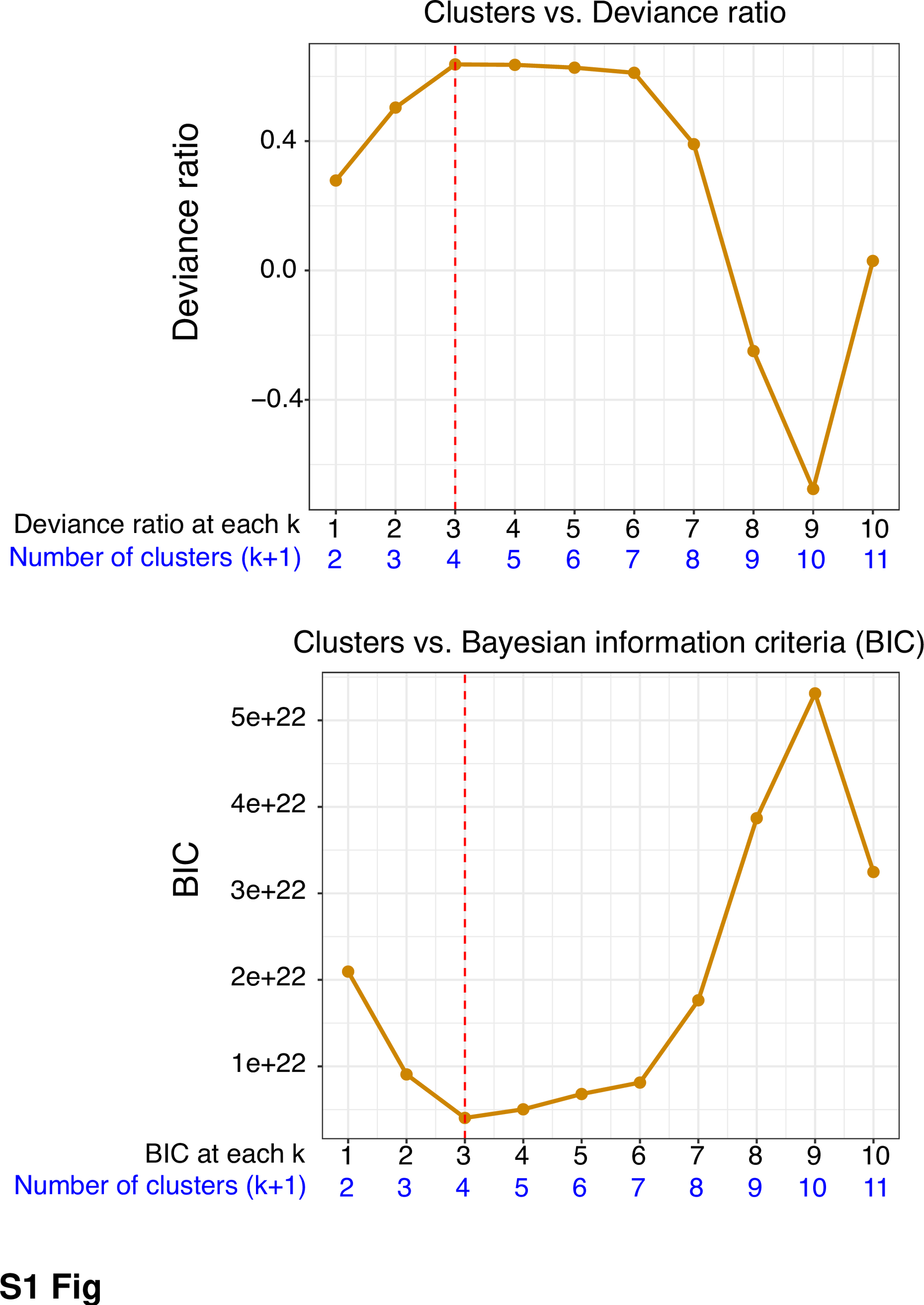

Supplement: S1 Fig — The deviance ratio (top) and the Bayesian information criterion—BIC (bottom) at each K (number of eigen features). The optimal solution is observed with K = 3 when the BIC is the minimum and the deviance ratio is the maximum dividing our samples into 4 clusters (k+1). (TIF) [file pbio.3002607.s001.tif]

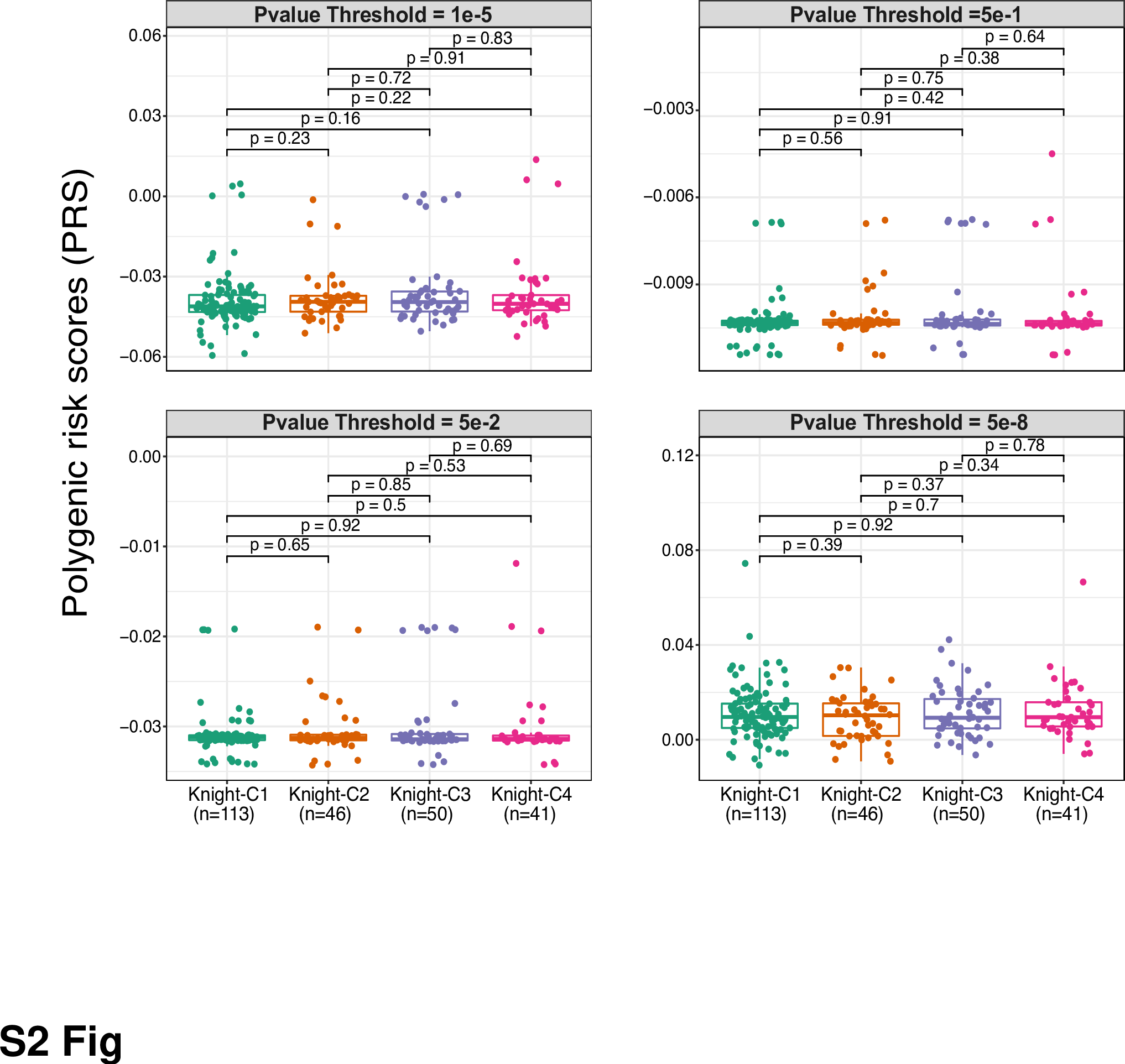

Supplement: S2 Fig — Boxplots showing the polygenic risk scores (PRS) across 4 clusters using 4 different p-value thresholds showing no significant association between Knight-C4 and PRS scores. The data underlying this figure can be found in S1 Data. (TIF) [file pbio.3002607.s002.tif]

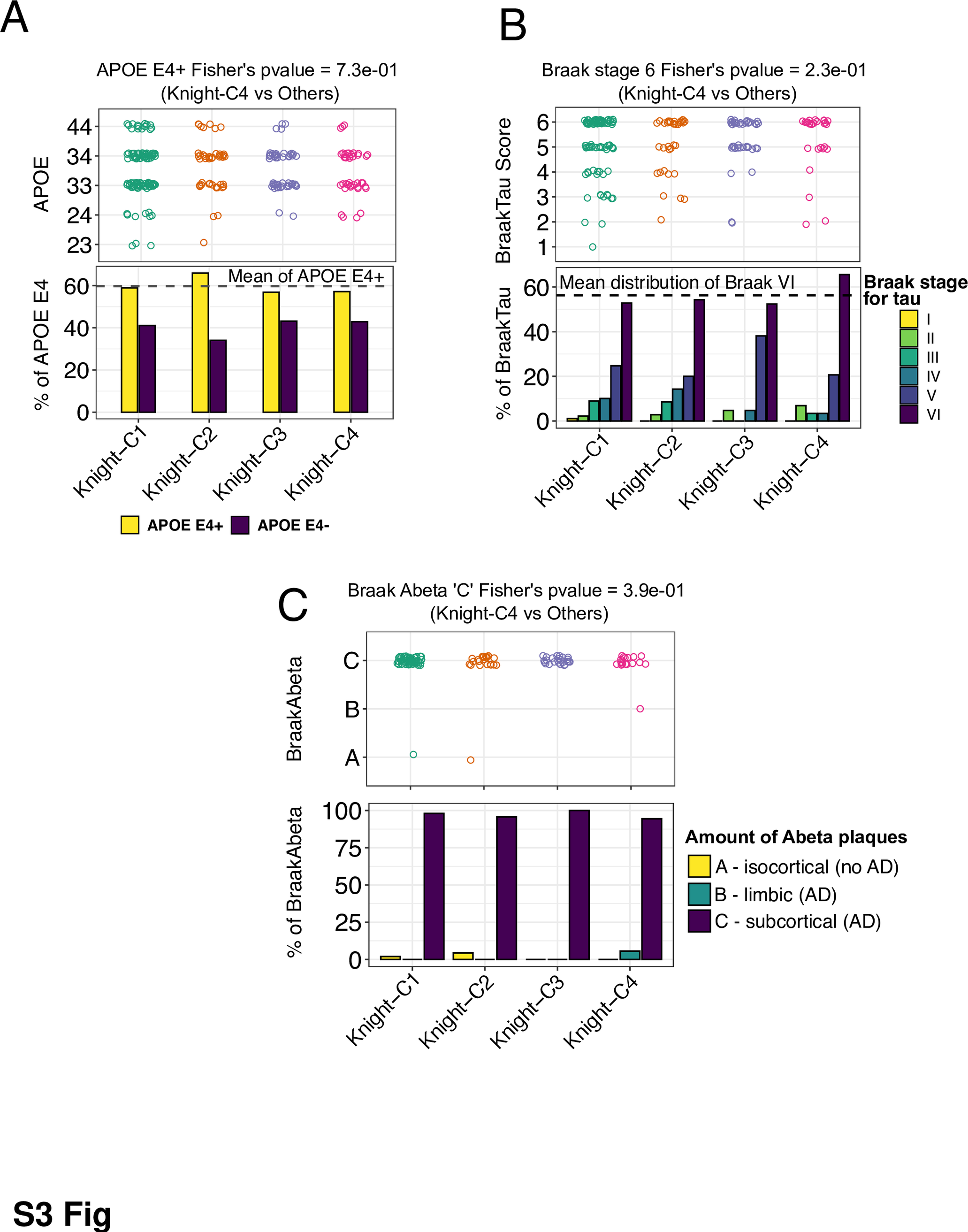

Supplement: S3 Fig — (A) The distribution of the APOE ℇ4 allele across 4 clusters shows no association with Knight-C4. (B) The distribution of Braak scores for tau across 4 clusters shows no significant correlation between Knight-C4 and Braak staging for tau. However, Knight-C4 exhibited more stage VI cases exceeding the mean distribution of Braak stage VI (dashed line) across all clusters. (C) The distribution of Braak scores for amyloid-β across 4 clusters showing no association with Knight-C4. The data underlying this figure can be found in S1 Data. (TIF) [file pbio.3002607.s003.tif]

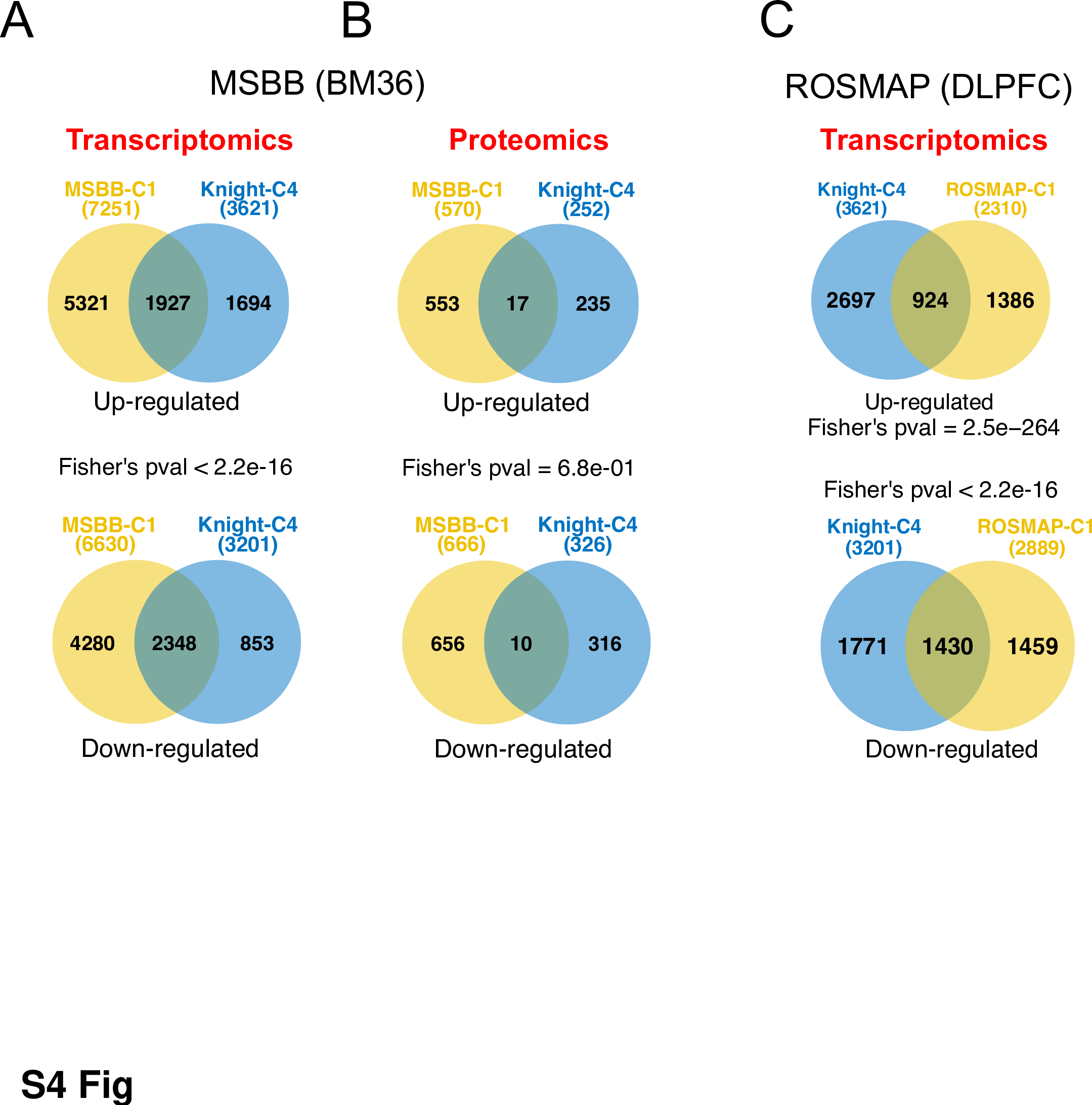

Supplement: S4 Fig — (A) Venn diagrams depicting the overlap between significant genes in Knight-C4 and MSBB-C1 cohorts, showing a replication of the molecular signature associated with worse cognitive outcomes. (B) Same as “A” but for significant proteins. (C) Venn diagrams depicting the overlap between significant genes in Knight-C4 and ROSMAP-C1 cohorts, showing a replication of the molecular signature associated with worse cognitive outcomes. (TIF) [file pbio.3002607.s004.tif]

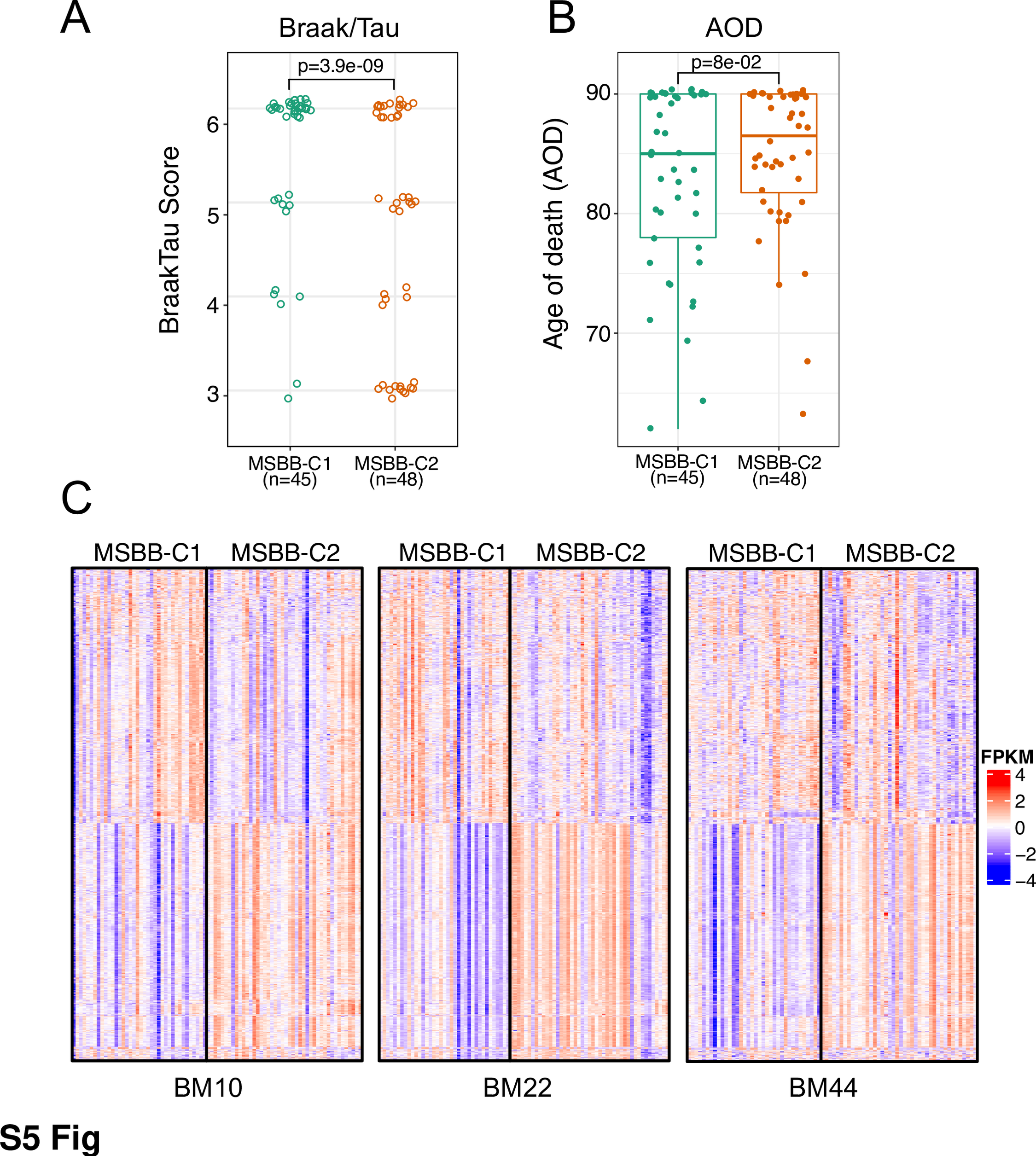

Supplement: S5 Fig — (A) Boxplots showing MSBB-C1 are associated with high BraakTau scores. (B) Boxplots show a clear pattern of an association with the early age of death for cases in MSBB-C1. (C) Heatmaps showing the replication of transcriptomic profiles of MSBB-C1 from the BM36 region in additional regions including frontal pole (BM10), superior temporal gyrus (BM22), and inferior frontal gyrus (BM44) brain regions from the MSBB study. The data underlying panels A and B can be found in S1 Data. (TIF) [file pbio.3002607.s005.tif]

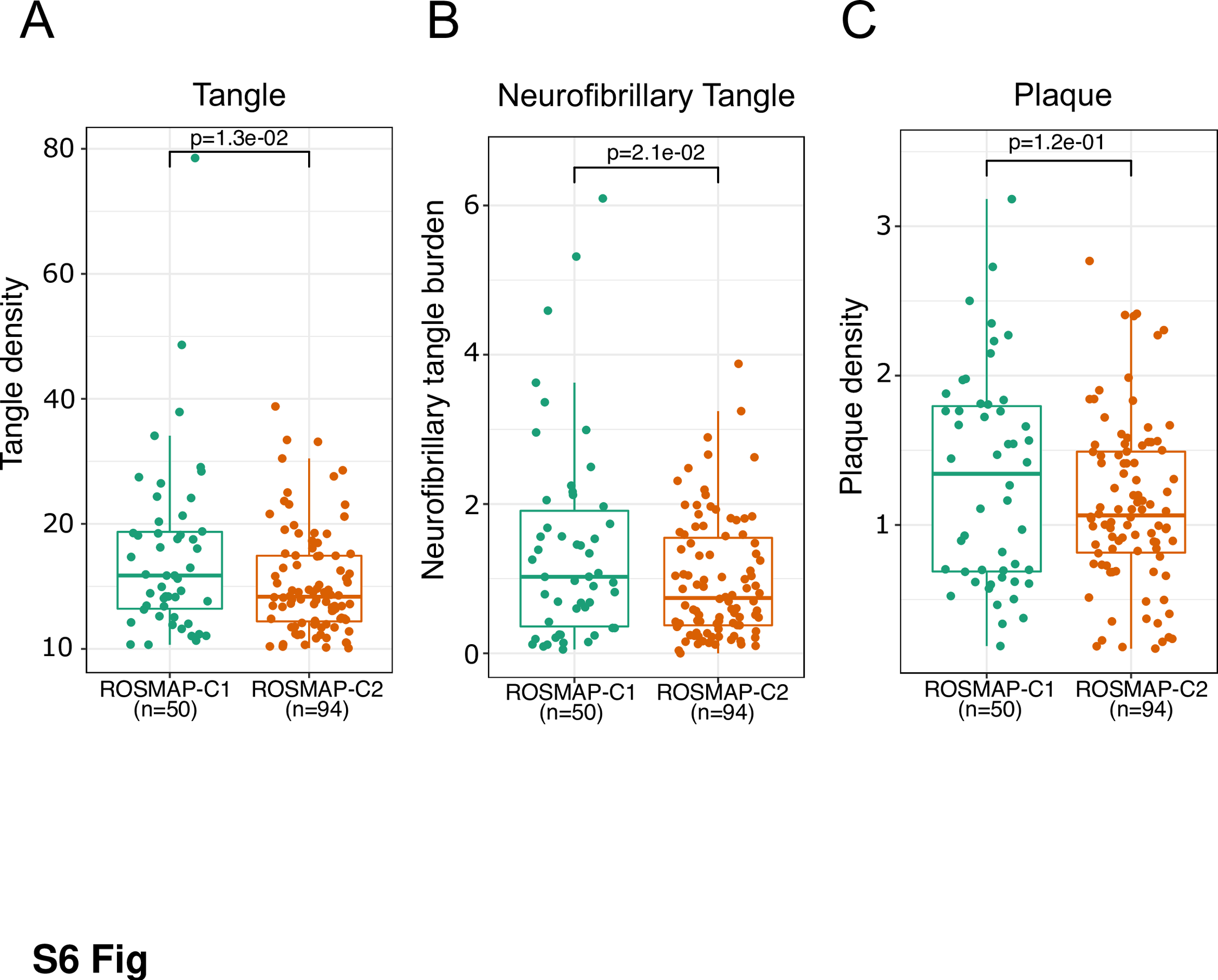

Supplement: S6 Fig — (A) Boxplots showing ROSMAP-C1 association with high tangle densities. (B) Boxplots showing ROSMAP-C1 association with neurofibrillary tangle burdens. (C) Boxplots showing higher plaque densities for AD cases in ROSMAP-C1. The data underlying this figure can be found in S1 Data. (TIF) [file pbio.3002607.s006.tif]

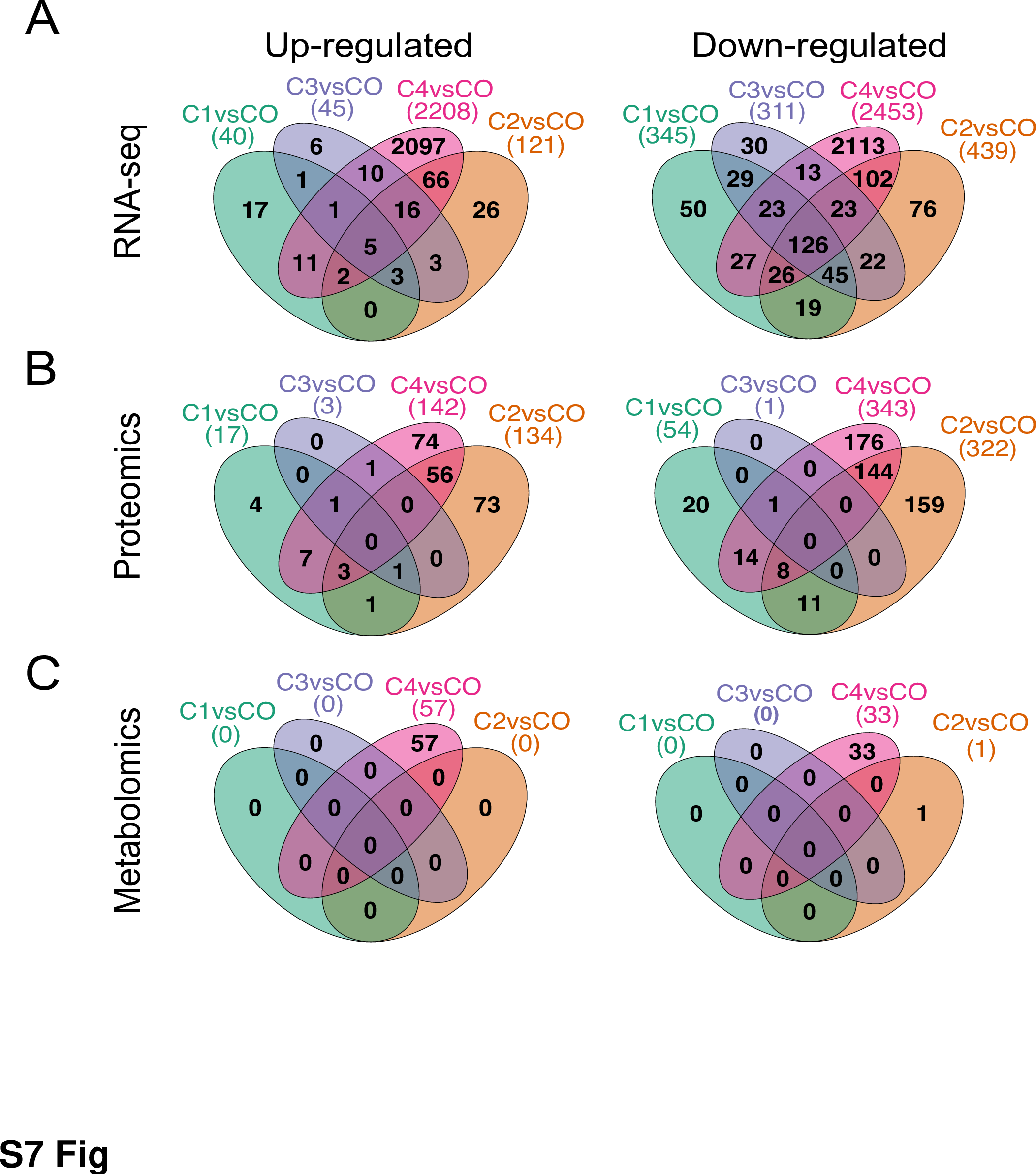

Supplement: S7 Fig — (A) Venn diagrams show the differentially expressed genes detected in each cluster (cluster vs. control). (B) Same as panel “A” but for proteomics. (C) Same as panel “A” but for metabolomics. (TIF) [file pbio.3002607.s007.tif]

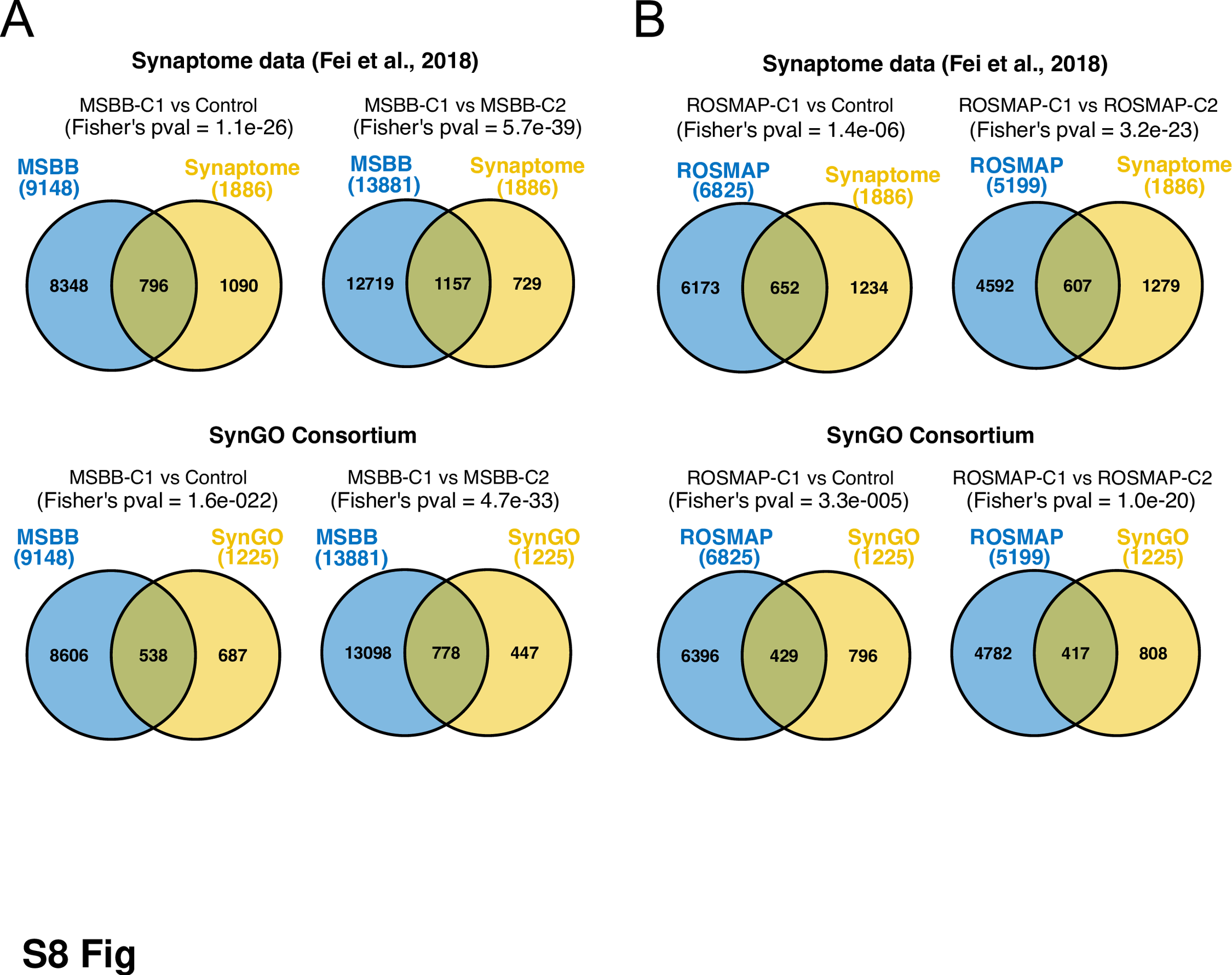

Supplement: S8 Fig — (A) Venn Diagrams showing the overlap between significant genes in MSBB-C1 and synaptic genes from Fei and colleagues [46] and SynGO [47] datasets. (B) Same as “A” but overlaps with significant genes in ROSMAP-C1. (TIF) [file pbio.3002607.s008.tif]

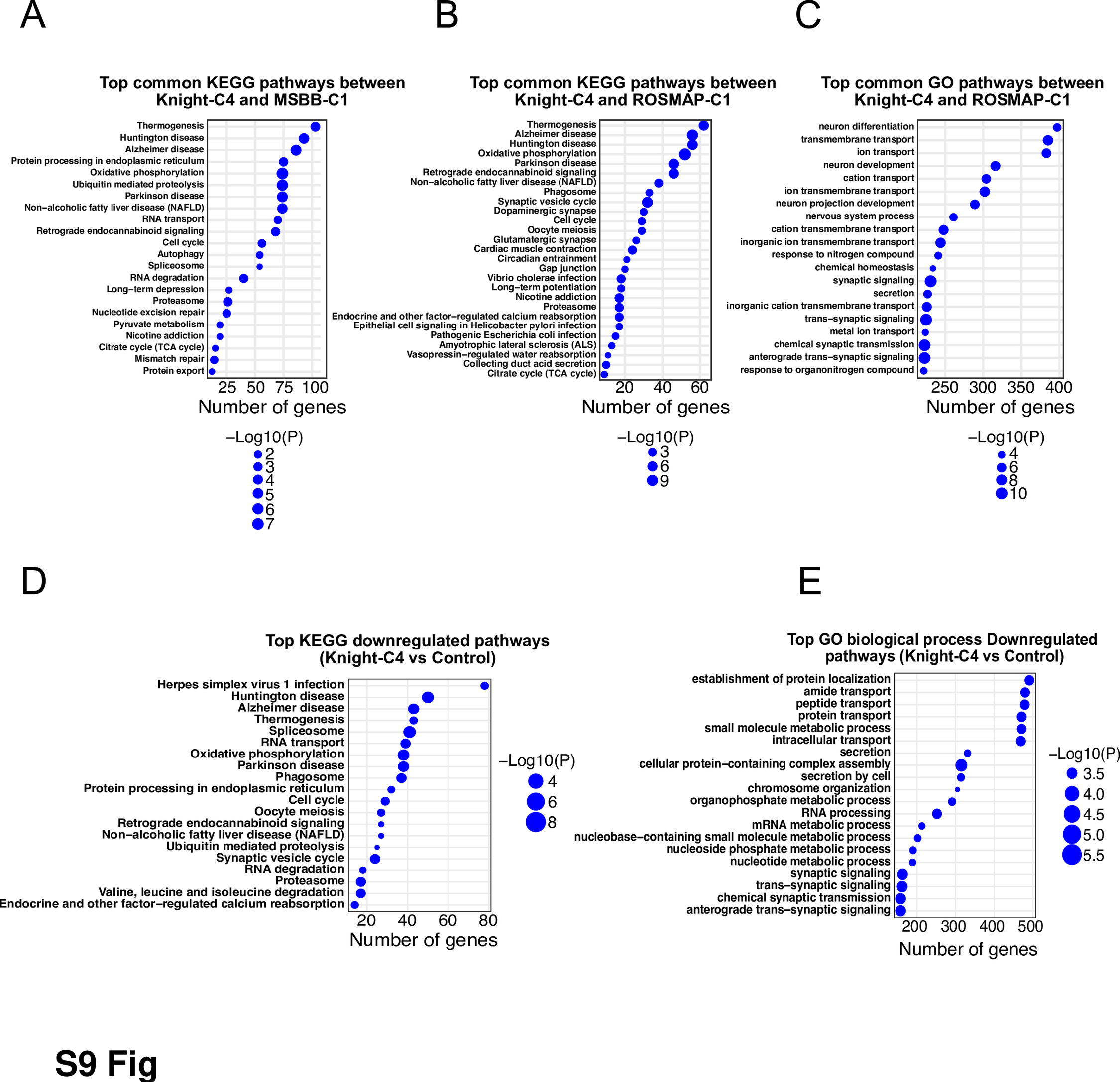

Supplement: S9 Fig — (A). Top common KEGG pathways dysregulated in Knight-C4 and MSBB-C1 (Cluster vs. other AD cases). (B) Top common KEGG pathways dysregulated in Knight-C4 and ROSMAP-C1 (Cluster vs. other AD cases). (C) Top common GO biological process pathways dysregulated in Knight-C4 and ROSMAP-C1 (Cluster vs. other AD cases). (D) Top 20 KEGG pathways enriched in Knight-C4 down-regulated genes (Knight-C4 vs. control). (E) Top 20 GO biological process pathways enriched in Knight-C4 down-regulated genes (Knight-C4 vs. control). (TIF) [file pbio.3002607.s009.tif]

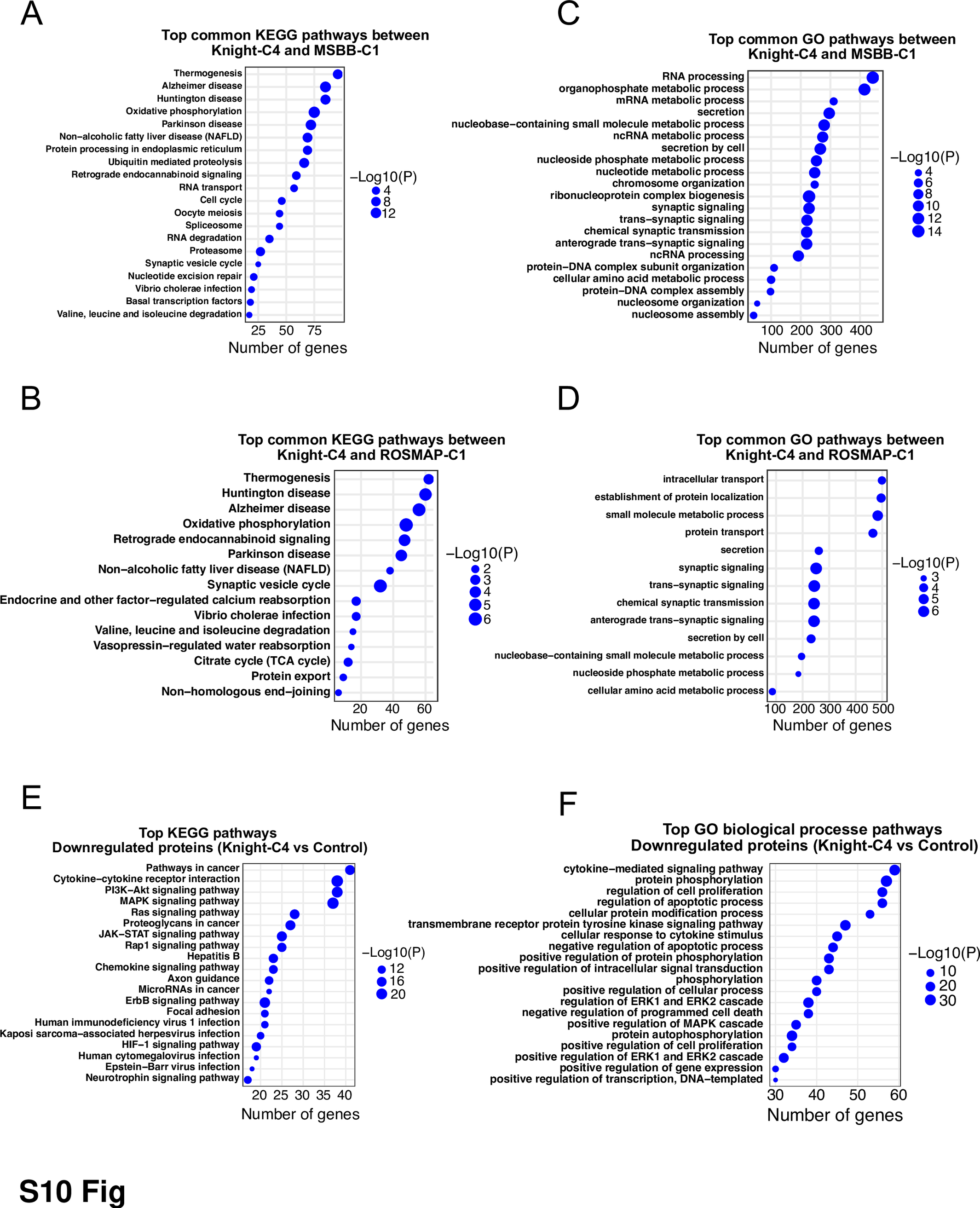

Supplement: S10 Fig — (A) Top common KEGG pathways dysregulated in Knight-C4 and MSBB-C1. (B) Top common KEGG pathways dysregulated in Knight-C4 and ROSMAP-C1. (C) Top common GO biological process pathways dysregulated in Knight-C4 and MSBB-C1. (D) Top common GO biological process pathways dysregulated in Knight-C4 and ROSMAP-C1. (E) Top 20 KEGG pathways associated with the down-regulation of proteins in Knight-C4. (F) Top 20 GO biological process pathways enriched in Knight-C4 down-regulated proteins. (TIF) [file pbio.3002607.s010.tif]

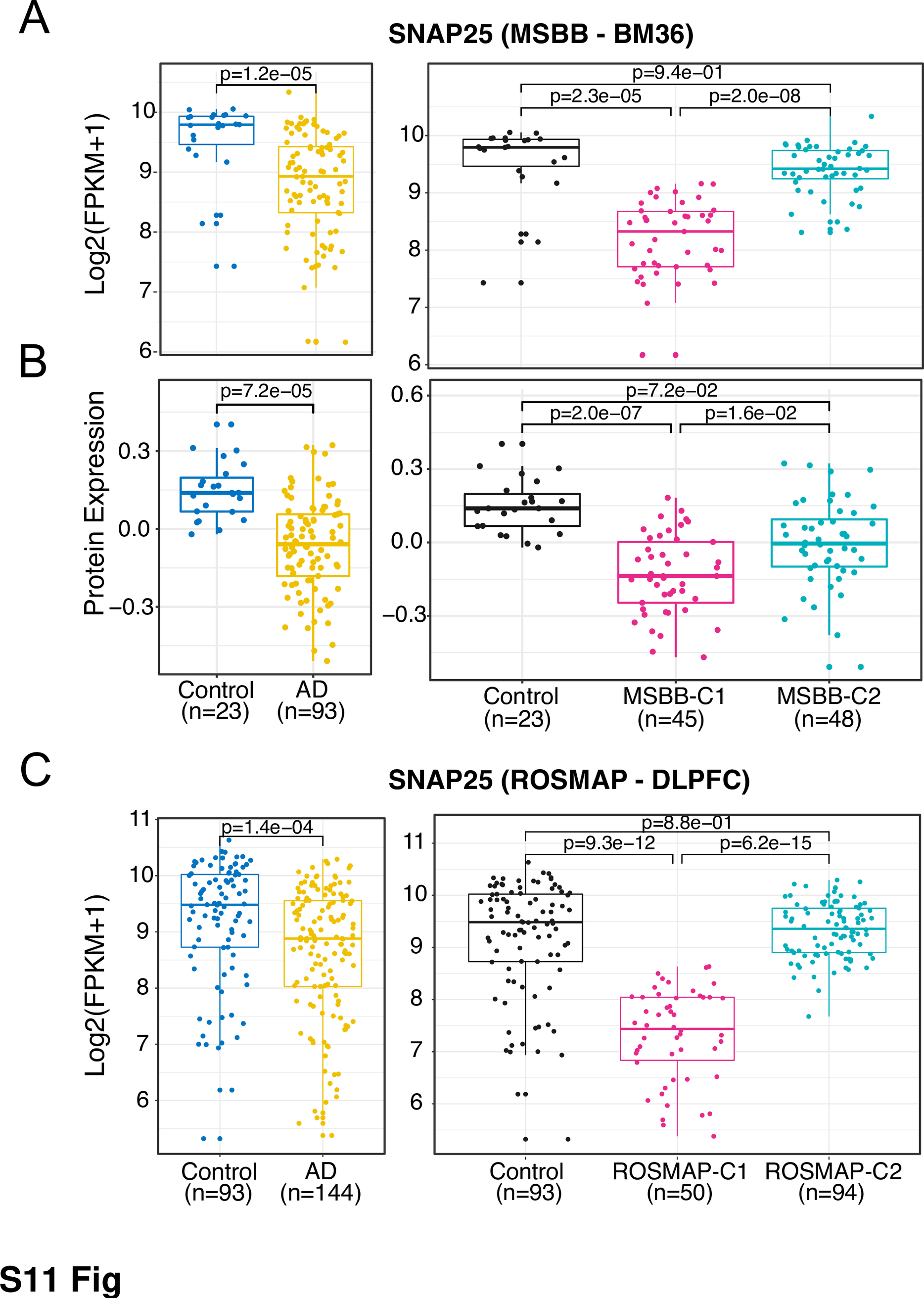

Supplement: S11 Fig — (A) Boxplots showing transcriptomic profiles of SNAP25 across the 2 clusters (right) and all ADs (left) in the MSBB (BM36) cohort. (B) Boxplots showing proteomic (TMT) profiles of SNAP25 across the 2 clusters as mentioned in “A.” (C) Boxplots showing transcriptomic profiles of SNAP25 across the 2 clusters (right) and all ADs (left) in ROSMAP (DLPFC) cohort. The data underlying this figure can be found in S1 Data. (TIF) [file pbio.3002607.s011.tif]

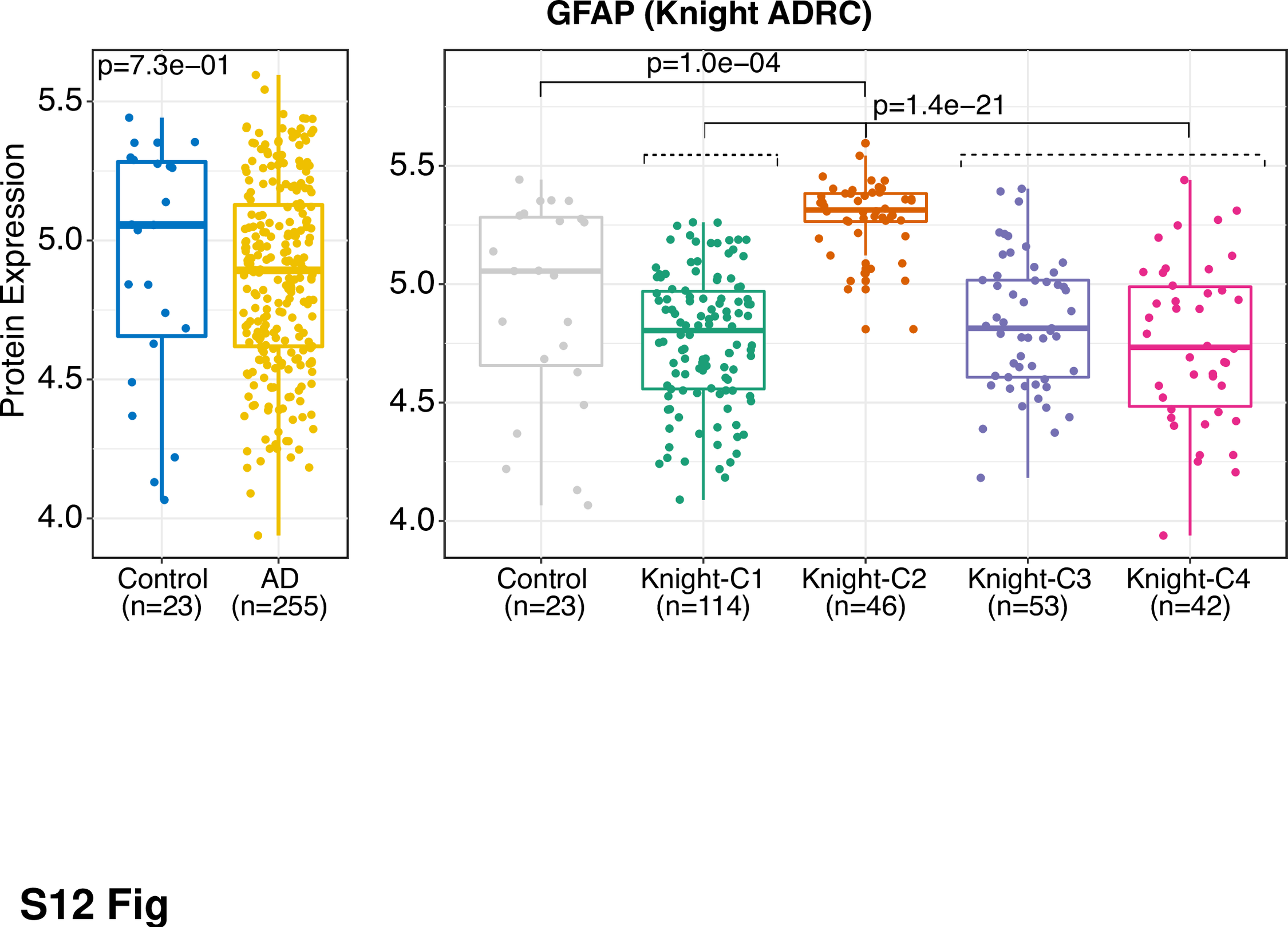

Supplement: S12 Fig — GFAP protomeric levels are significantly increased in Knight-C2 compared to the control and other AD cases (right). The data underlying this figure can be found in S1 Data. (TIF) [file pbio.3002607.s012.tif]

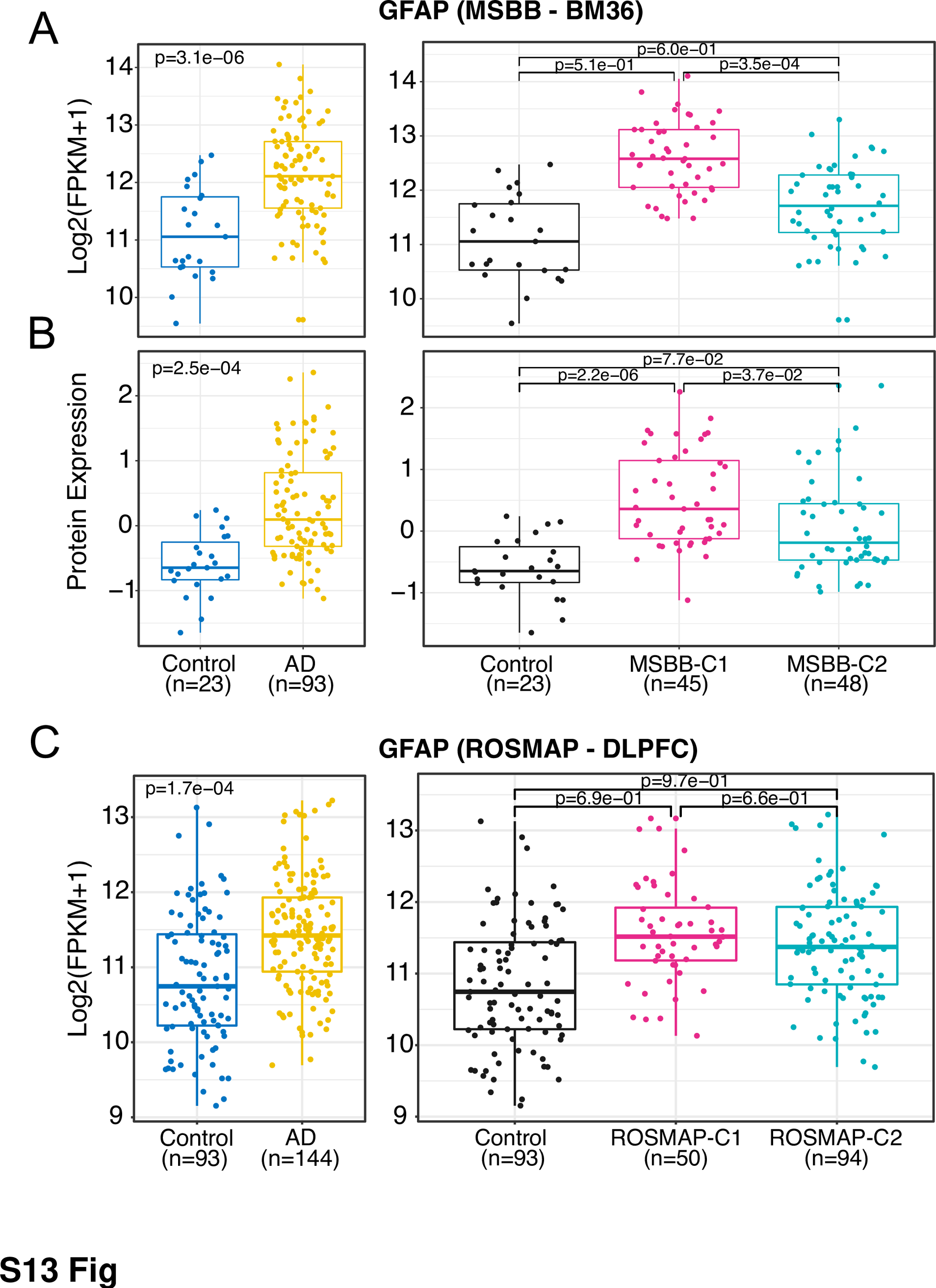

Supplement: S13 Fig — (A) Boxplots showing the transcriptomic profiles of GFAP across the 2 clusters (right) and all AD cases (left) in the MSBB (BM36) cohort. (B) Same as “A” but for the proteomic profiles (TMT). (C) Same as “A” but in ROSMAP (DLPFC) cohort. The data underlying this figure can be found in S1 Data. (TIF) [file pbio.3002607.s013.tif]

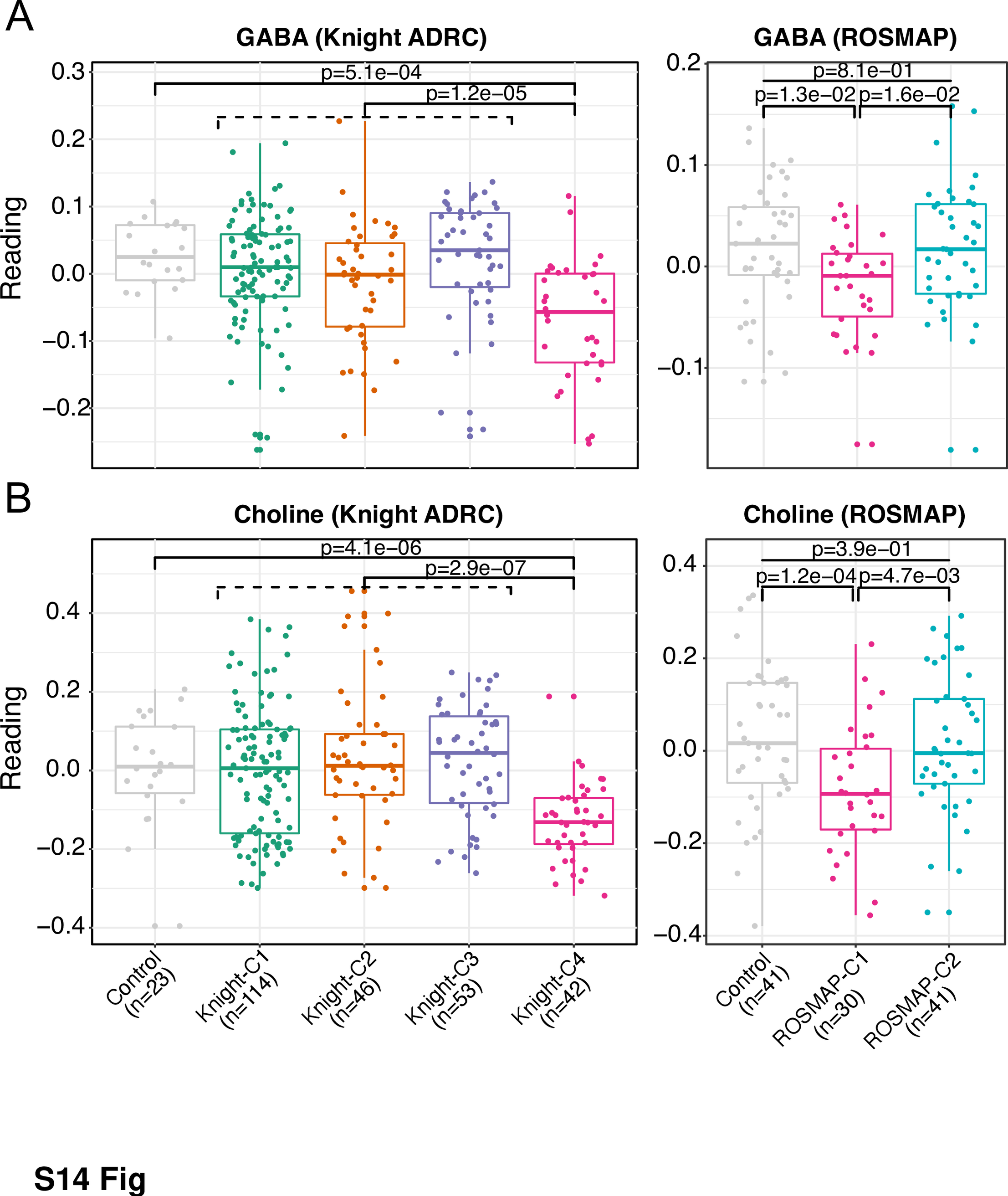

Supplement: S14 Fig — (A) Boxplots showing the decrease of metabolomics profiles of gamma-aminobutyrate (GABA) in Knight-C4 (left) and ROSMAP-C1 (right). (B) Same as “A” but for choline metabolite. The data underlying this figure can be found in S1 Data. (TIF) [file pbio.3002607.s014.tif]

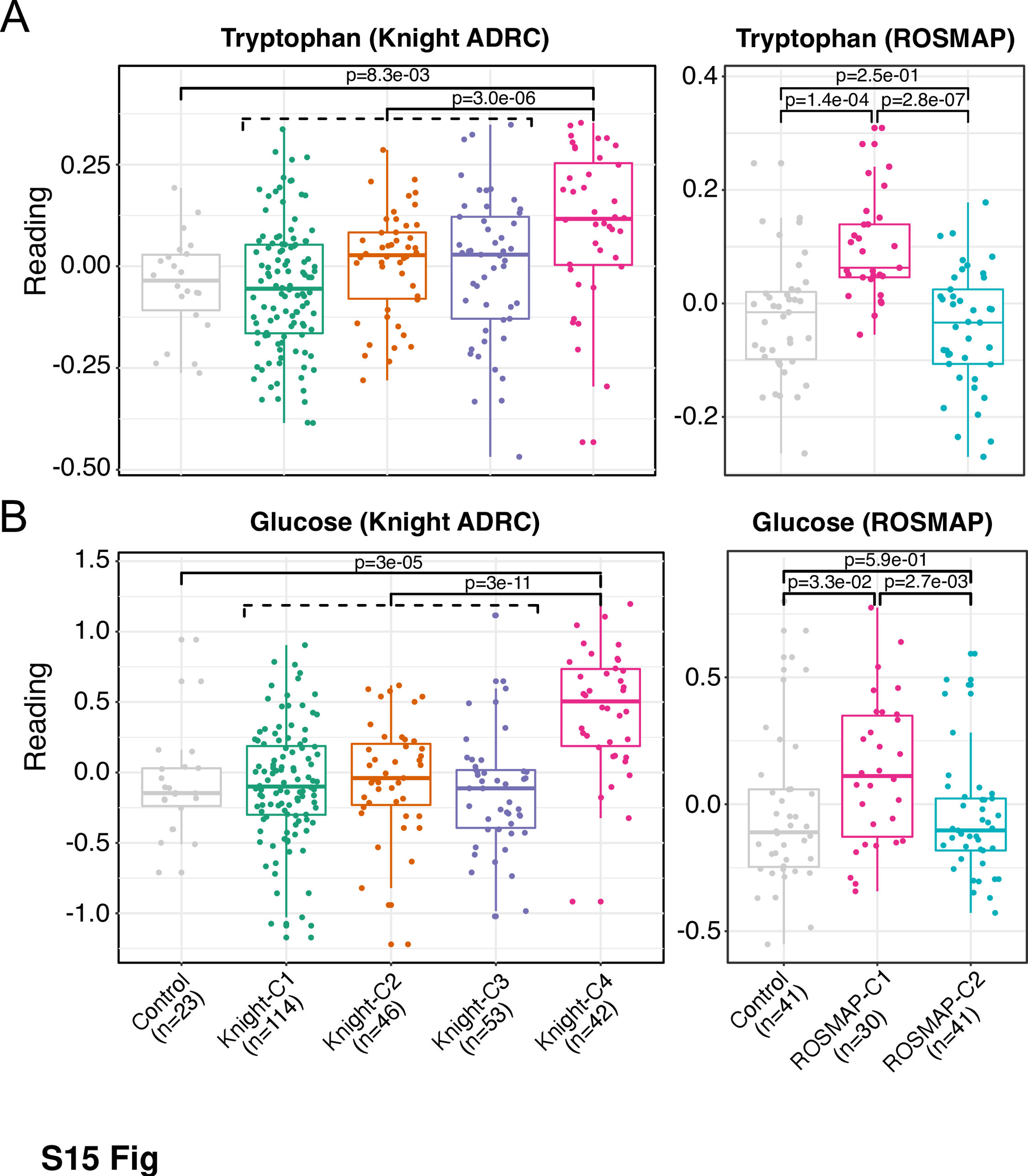

Supplement: S15 Fig — (A) Boxplots showing the increase of metabolomic profiles of tryptophan in Knight-C4 (left) and ROSMAP-C1 (right). (B) Same as “A” but for glucose metabolite. The data underlying this figure can be found in S1 Data. (TIF) [file pbio.3002607.s015.tif]

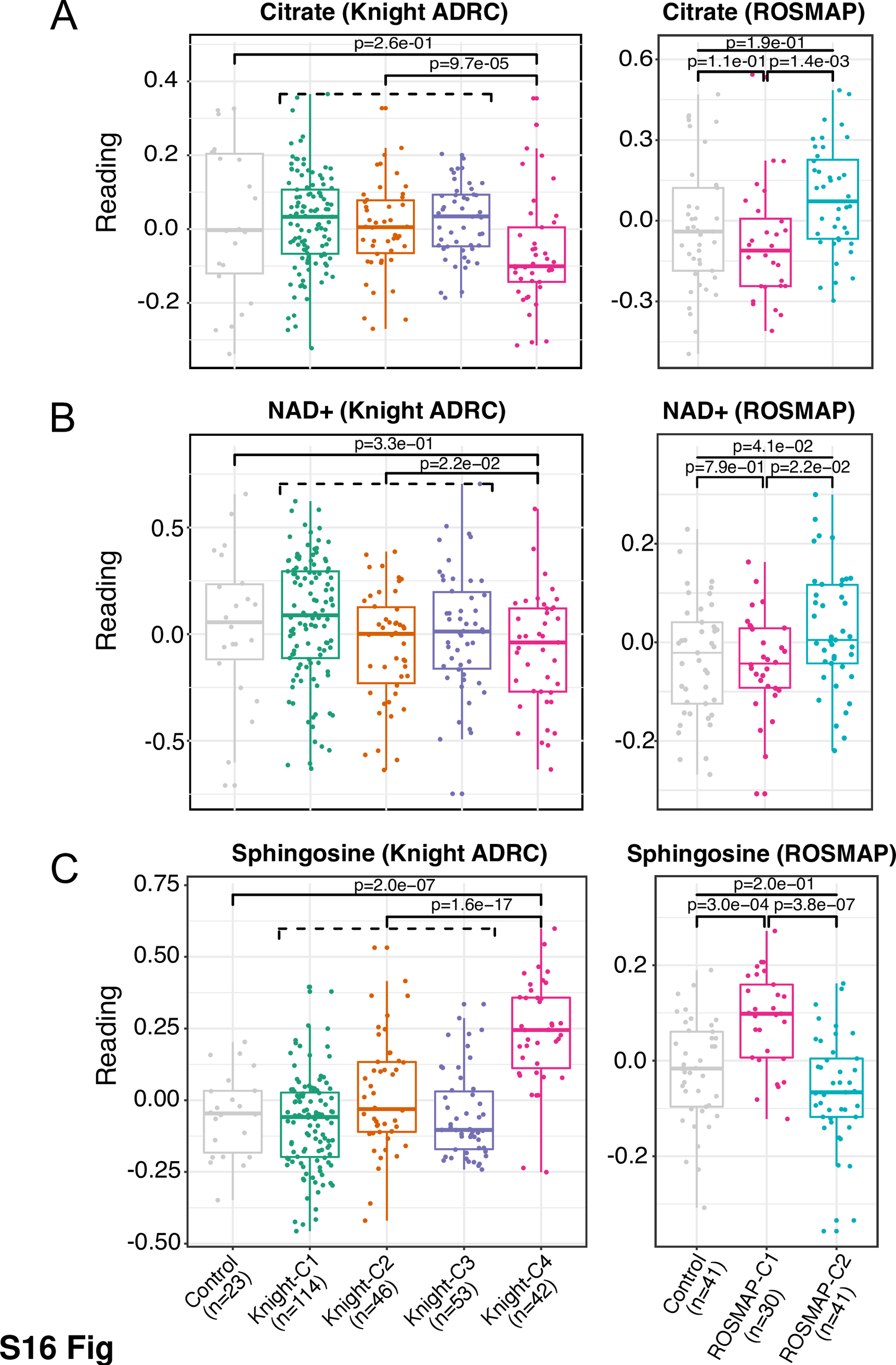

Supplement: S16 Fig — (A) Boxplots showing the decrease of metabolomic profiles of citrate in Knight-C4 (left) and ROSMAP-C1. (B) Boxplots showing the decrease of metabolomic profiles of NAD+ in both Knight-C4 (left) and ROSMAP-C1 (right). (C) Boxplots showing the increased metabolism levels of sphingosine in both Knight-C4 (left) and ROSMAP-C1 (right). The data underlying this figure can be found in S1 Data. (TIF) [file pbio.3002607.s016.tif]

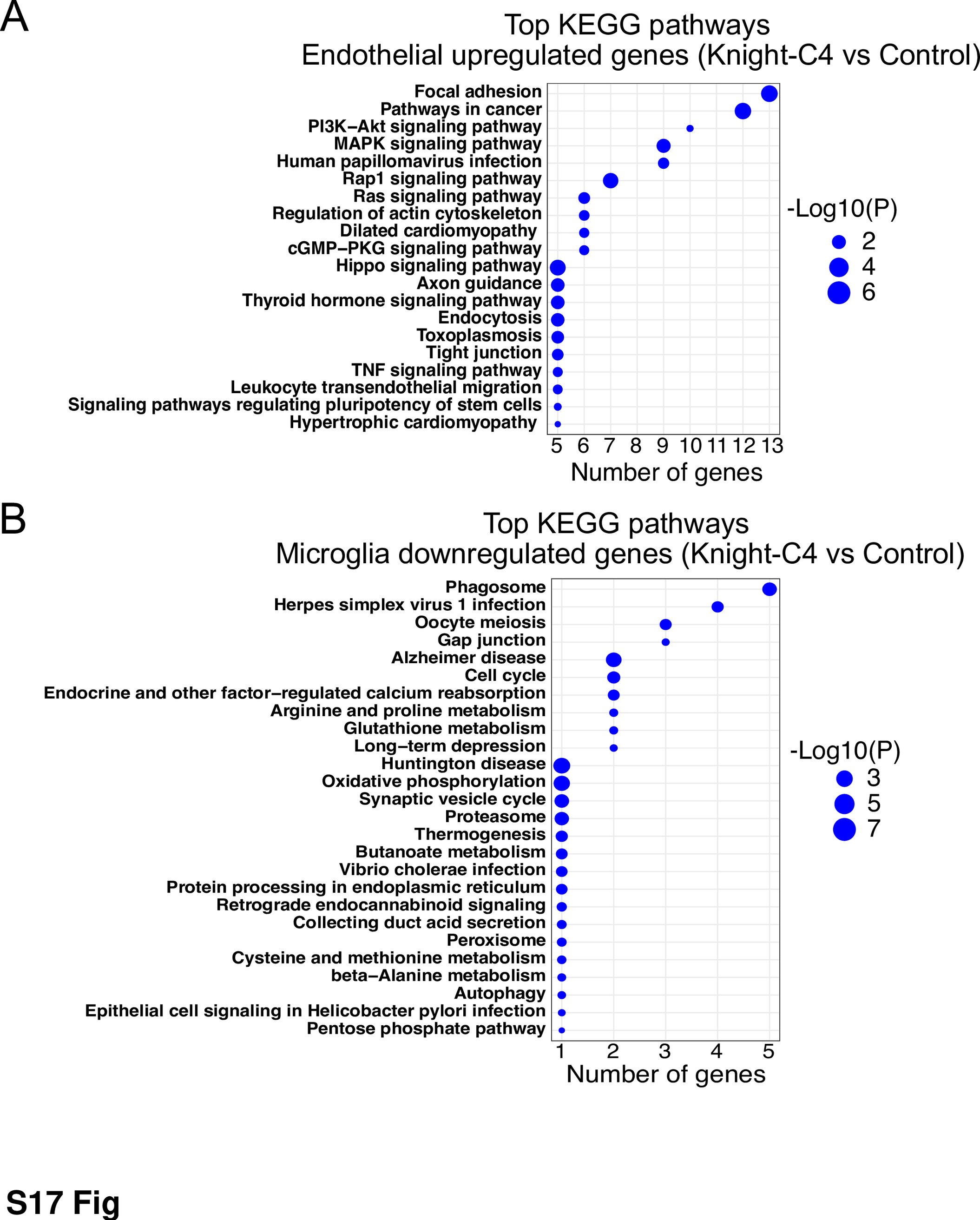

Supplement: S17 Fig — (A) Top 20 KEGG pathways enriched in endothelial up-regulated genes (Knight-C4 vs. Control). (B) Top 20 KEGG pathways enriched in microglia down-regulated genes (Knight-C4 vs. Control). (TIF) [file pbio.3002607.s017.tif]

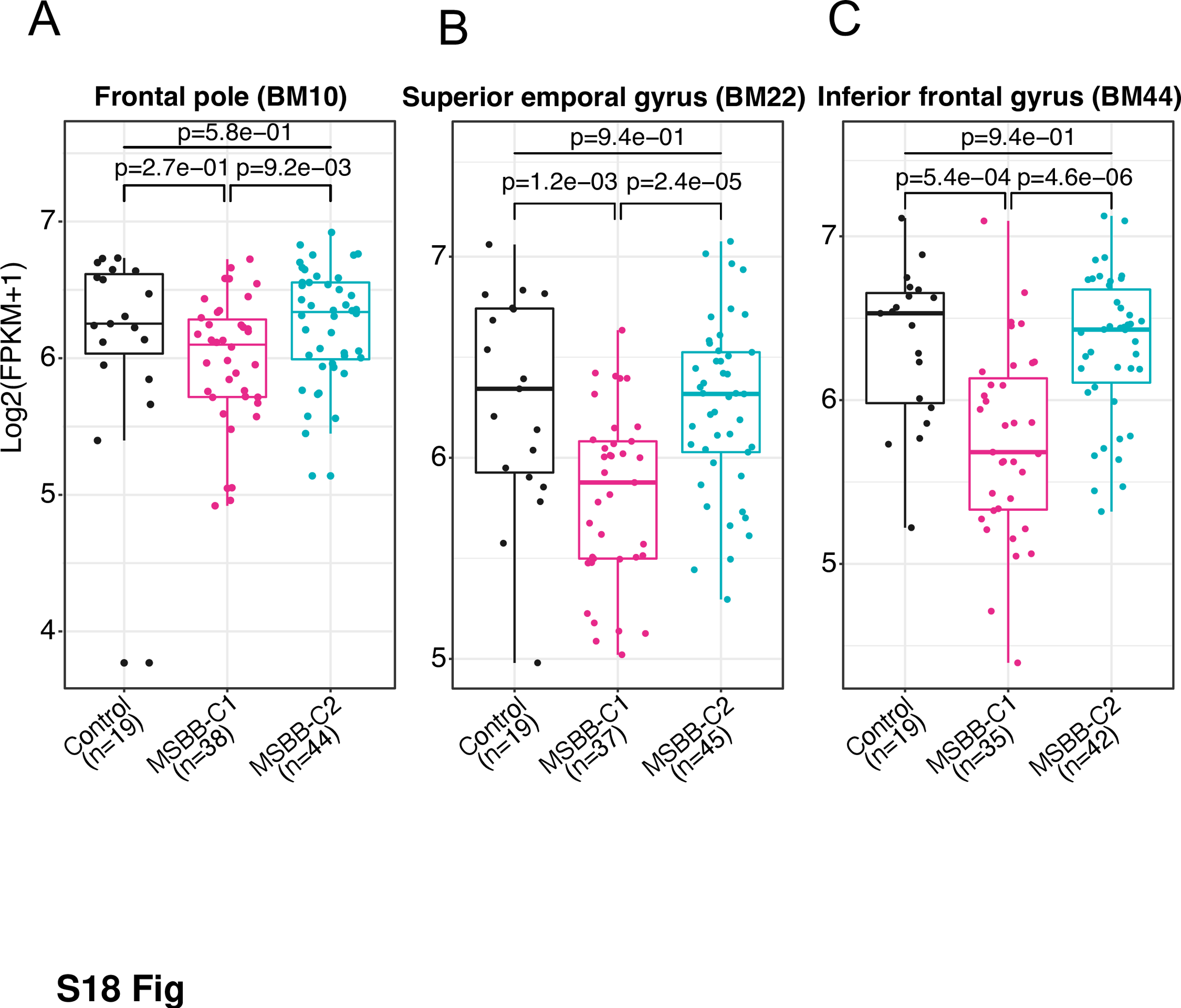

Supplement: S18 Fig — (A) Boxplot showing the transcriptomic profiles of SNCA in MSBB-C1 from the frontal pole (BM10) brain region. (B) Same as “A” but from superior temporal gyrus (BM22). (C) Same as “A” and “B” but from the inferior frontal gyrus (BM44) brain region from MSBB. The data underlying this figure can be found in S1 Data. (TIF) [file pbio.3002607.s018.tif]

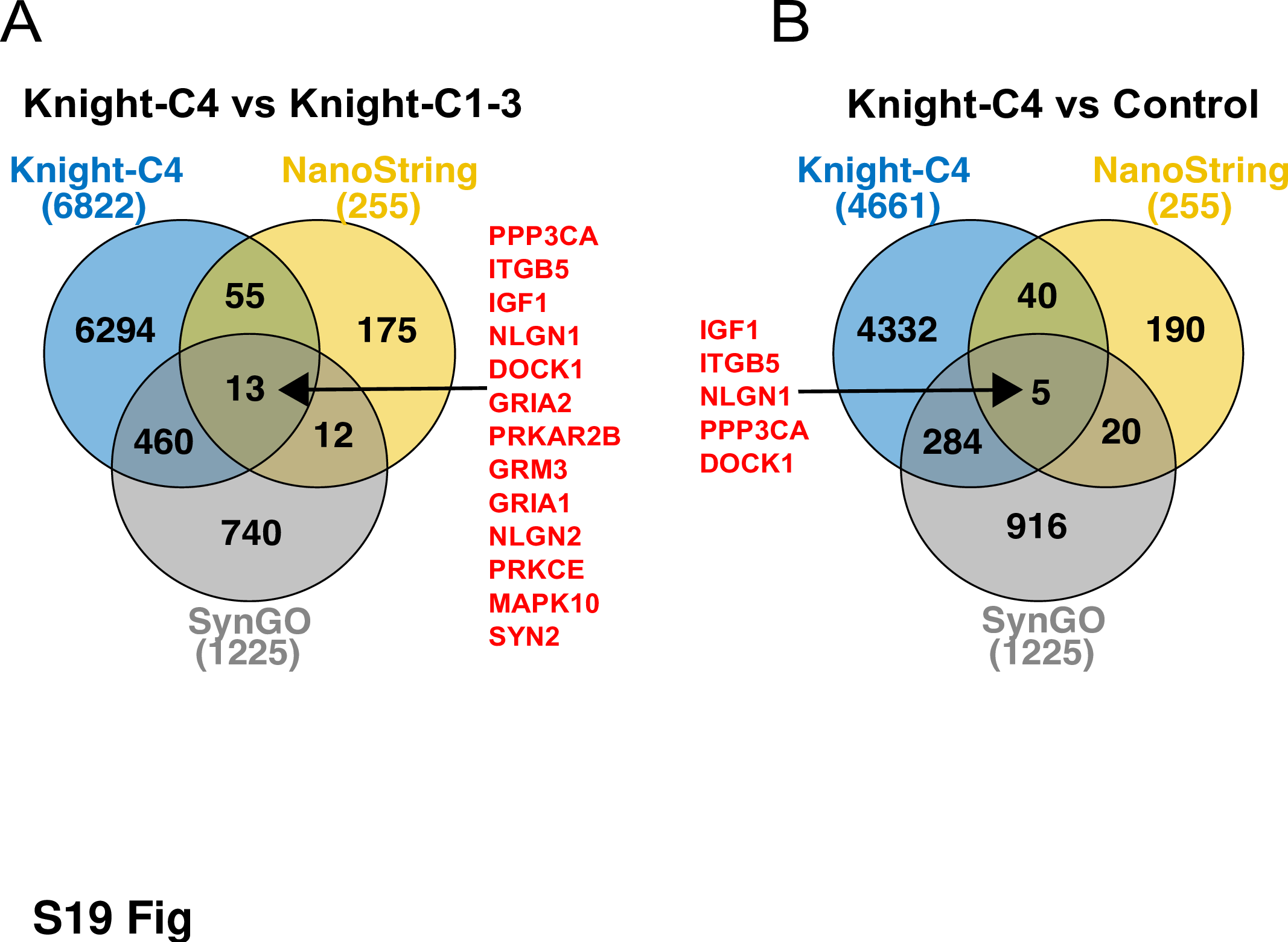

Supplement: S19 Fig — (A) Venn diagram showing the overlap between significant genes in Knight-C4 compared to other clusters, significant genes from NanoString gene expression data from A53T αSyn mouse models, and synaptic genes from SynGO dataset. (B) Same as “A” but using significant genes in Knight-C4 compared to the control. (TIF) [file pbio.3002607.s019.tif]

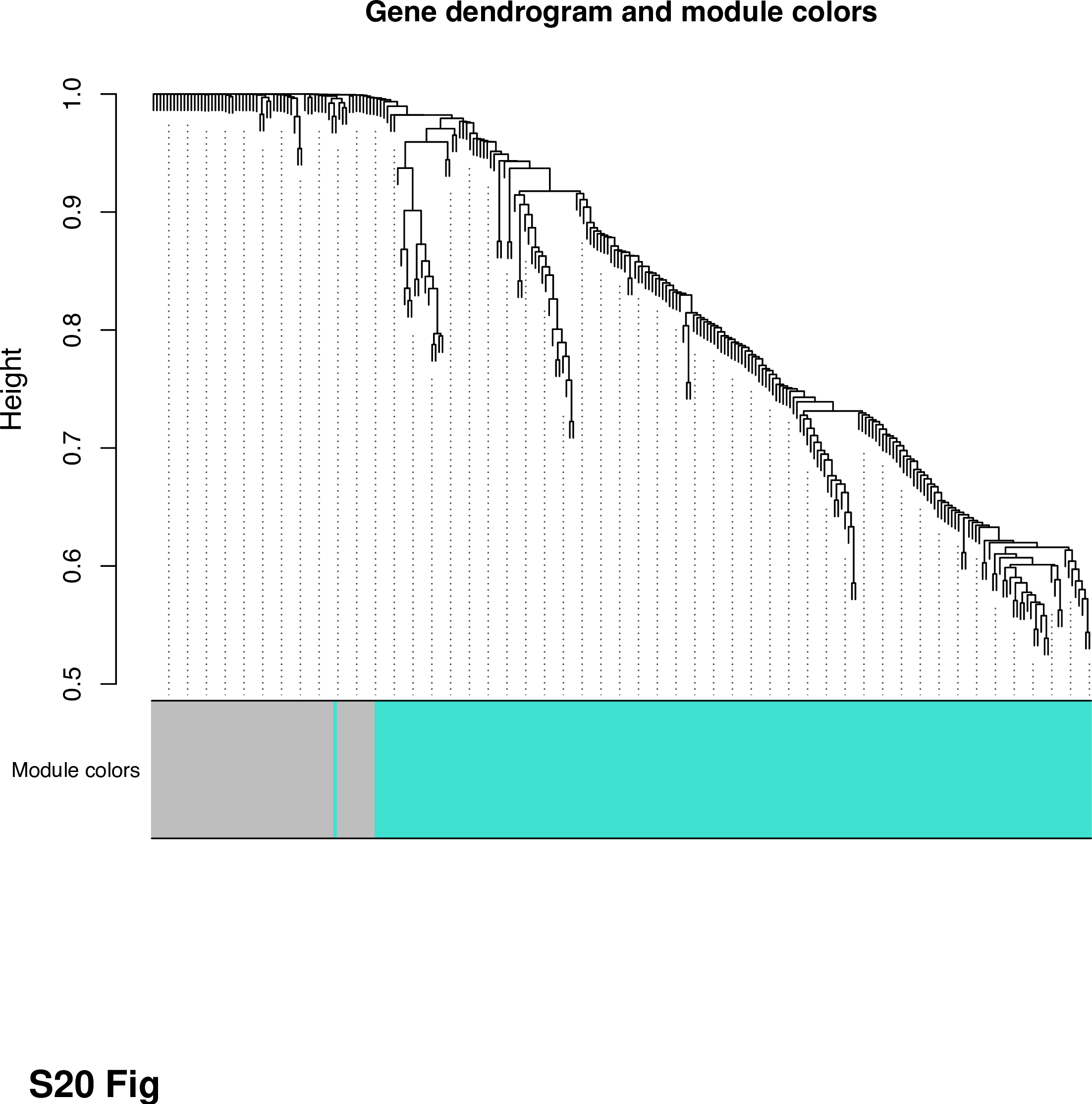

Supplement: S20 Fig — (TIF) [file pbio.3002607.s020.tif]

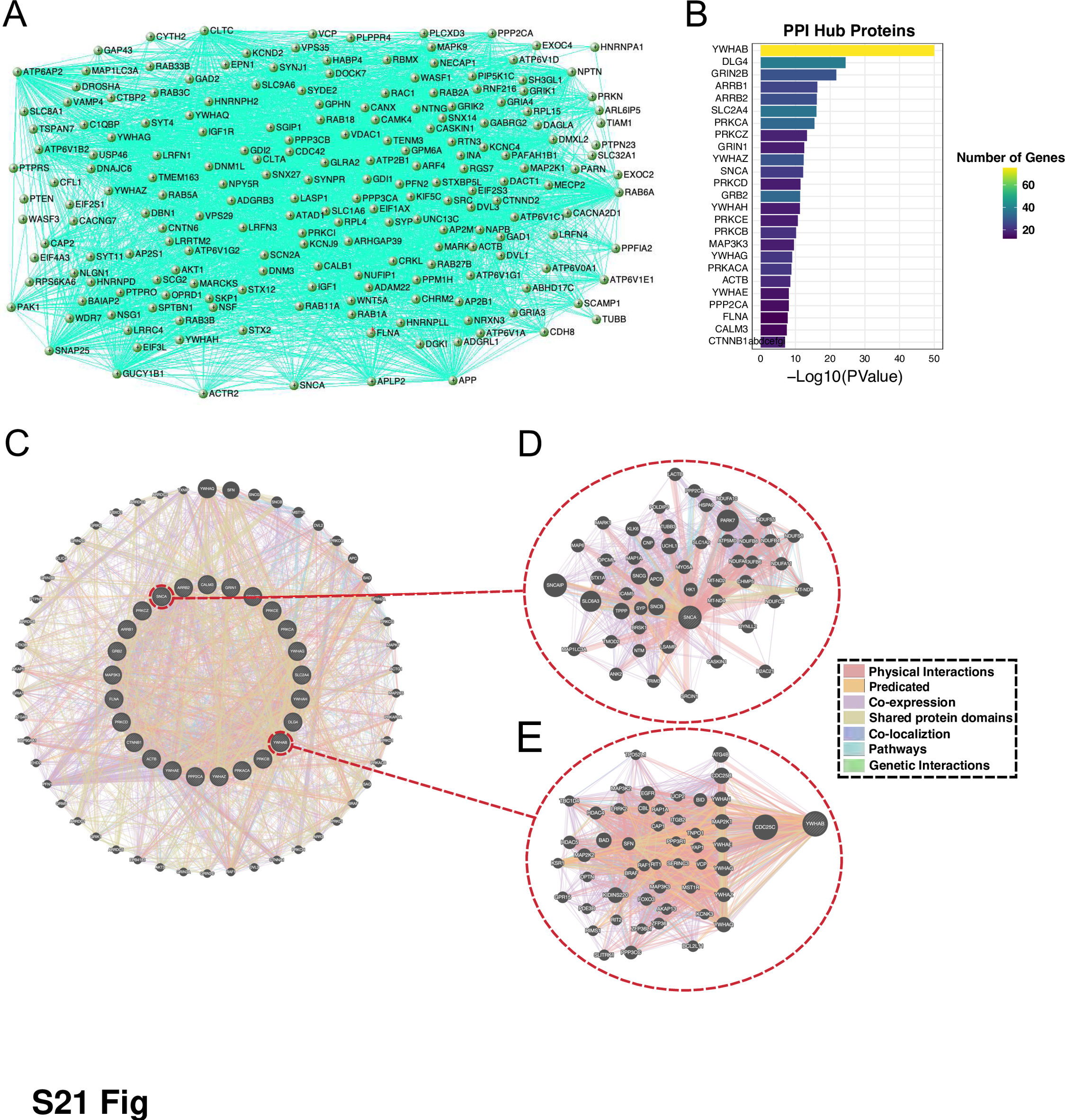

Supplement: S21 Fig — (A) The gene interaction network for the turquoise module shows high interactions between dysregulated synaptic genes such as SNAP25, SNCA, and APP. (B) Barplot showing the top 25 hub proteins extracted from protein–protein interaction (PPI) hub proteins pathways. (C) Gene interaction network showing the relatedness of the 25 dysregulated hub proteins. The plot demonstrates multiple strong interactions including physical and genetic interactions among the query genes (25 hub proteins) and other genes. (D) YWHAB sub-network shows the genes highly connected with YWHAB, some of which are examples of either synaptic or other AD-related genes. (E) Same as “D” but for SNCA sub-network. (TIF) [file pbio.3002607.s021.tif]

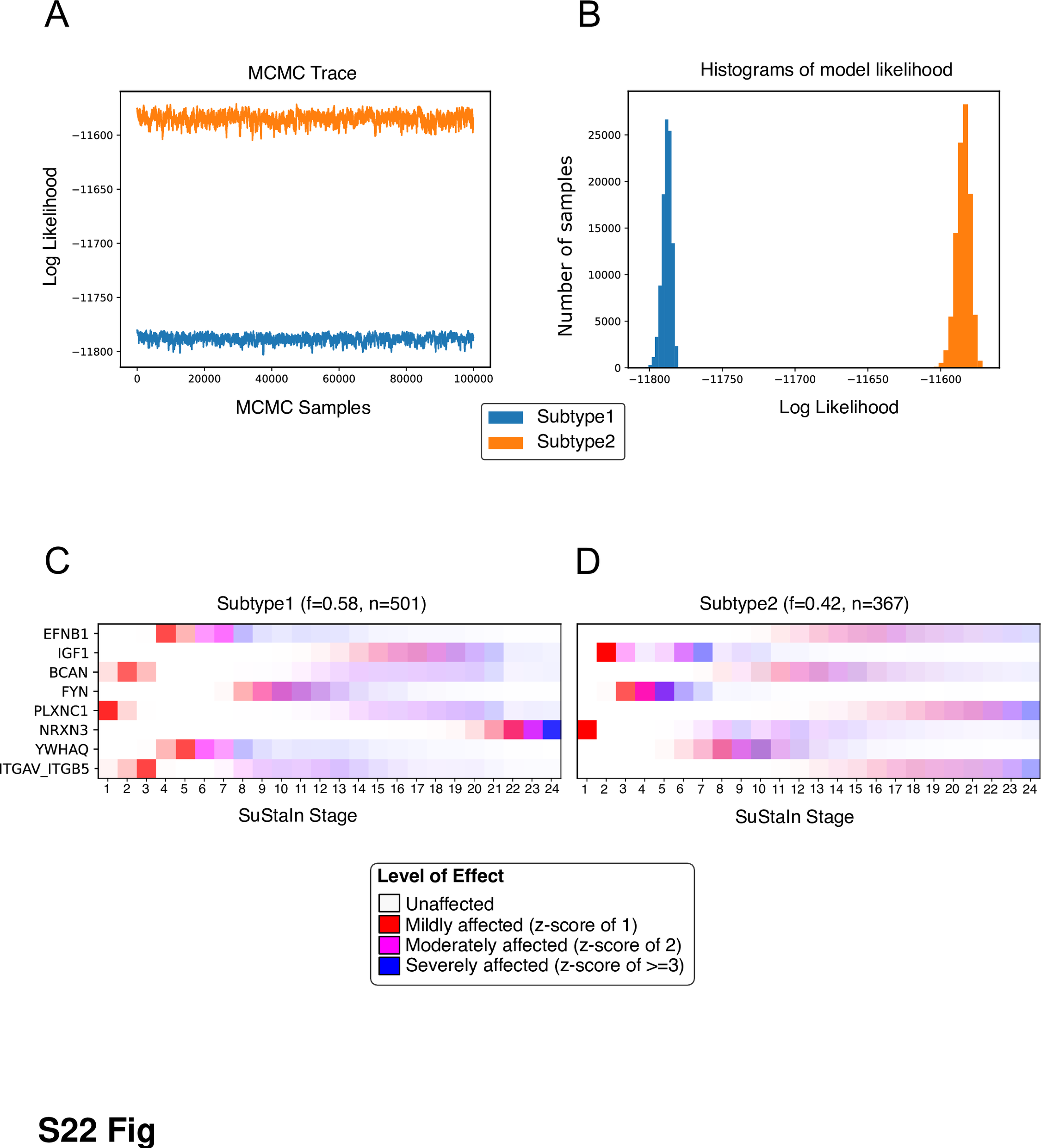

Supplement: S22 Fig — (A) Markov chain Monte Carlo (MCMC) trace generated by SuStaIn showing periodic patterns of the likelihood among MCMC samples in each subtype. (B) Histograms of the model likelihood generated by SuStaIn showing a model with a clear 2 subtypes. (C) Positional variance diagrams showing the progression patterns (SuStaIn stages) of Subtype1 for the 8 CSF biomarkers. The colors in each stage represent the effect on the CSF biomarker levels where white is unaffected; red is mildly affected (z-score of 1); magenta is moderately affected (z-score of 2); and blue is severely affected (z-score of 3 or more). (D) Same as “C” but for Subtype2. (TIF) [file pbio.3002607.s022.tif]
