# Supplementary material for: Brain high-throughput multi-omics data reveal molecular heterogeneity in Alzheimer’s disease
Source: PLoS Biol. 2024 Apr 30;22(4):e3002607. doi: 10.1371/journal.pbio.3002607 (PMC11086901; doi:10.1371/journal.pbio.3002607)
Supplement: Attachment — Submitted filename: Rebuttal_Letter_PLoSBio.pdf [file pbio.3002607.s038.pdf]

Reviewer's Comments:

We thank the reviewers for their valuable comments and critiques. We are excited to learn the reviewers found our study interesting (Rev #1), important (Rev #2), and timely (Rev #3), and we agree with the reviewers that some aspects of our manuscript require additional explanation and investigation. We have addressed all these comments, critiques, and suggestions in this revised version of the manuscript, which has improved the quality of our manuscript. Please find below our point-by-point response to the reviewers' comments. Changes to the manuscript have been highlighted in a 'track-changes' version of the manuscript.

Reviewer #1:

This is a very interesting study proposing a multi-omics Alzheimer's disease stratification that is cross-validated across independent datasets. Several analyses for clarifying the biological distinctiveness of the identified subtypes were performed, including comparisons with snRNA, cellular profiles, clinical data, etc. While the manuscript presents an extensive amount of relevant analyses, it also has some major shortcomings that preclude its publication at the current state. Some of the limitations, as the first mentioned below, are also very well discussed in the "subtyping" or "stratification" field, with key recent methodological advances/solutions proposed that are unfortunately not considered here. Similarly, the literature review is quite restricted and lacks essential antecedent research.

We thank the reviewer for the critical and constructive feedback. Please find a point-by-point response below addressing their concerns.

Major comments:

- A critical point is that the subtyping was based on direct clustering on the omics data (the features), without controlling in any way for the participants' disease stages. As it has been shown (e.g., Young et al, 2018, Nat. Comms) two relevant "axes" of variability/heterogeneity coexist in every diseased population, one cause by the differences in diseases stages and other by disease subvariants. Trying to detect any of these "axes" independently without considering the other is a common error in many studies focused on progression and heterogeneity. The identified subtypes here are very likely contaminated with variability from having subjects at different stages of disease advance. It may be the case that they are primarily capturing subjects' proximity in the disease's long timeline, and not similarity in disease mechanisms or subvariants. The authors should consider this important point, i.e., how to disentangle between real underlying subtypes and data clusters potentially confounded by similarities in disease severity.

We appreciate the reviewer for bringing up this significant matter. We fully agree that it is important to control for disease stages and that genotypes should be considered. However, our study's main goal is not to generate subtypes of the disease, our study aimed at integrating multi-modal molecular data with demographic, clinical and neuropathological data to understand the

heterogeneity observed in multiple modalities of omics data from postmortem brains. We did not stratify participants by their cognitive status (e.g., CDR at death) nor pathological stage (e.g., Braak), as we aimed to evaluate whether subtle molecular differences among sporadic AD omics can provide hints for a granular staging of disease (quantitative molecular neuropathology). We want to emphasize that we intentionally avoid referring to these clusters throughout the manuscript as "subtypes" since we are focused on the study of the heterogeneity among sporadic ADs (non-carriers of pathological mutations) and identify emergent molecular properties. For this reason, we chose to call them "profiles" instead.

We agree with the reviewer that disease staging and subtyping are highly valuable, but it is outside the scope of our study. This will encompass the inclusion of data from multiple omic layers and brain regions for the same cohort of donors. Instead, our study identified and replicated molecular profiles in multiple cohorts that ascertained different cortical regions, but we agree that molecular subtyping would be highly valuable. We hope this work emphasizes the need for parallel simultaneous ascertainment of multiple omics in multiple brain region. In addition, we identified that molecular markers featuring some of the clusters could be used to track changes in living patients using CSF data, opening the new biomarkers anchored in the biology of AD identified in postmortem brain tissue.

Furthermore, we recognize the importance of disease staging and subtyping, thus, we employed the method (SuStaIn), suggested by the reviewer, which proved to uncover data-driven disease phenotypes with distinct temporal progression patterns to analyze proteomic data from the cerebrospinal fluid. We selected 8 CSF synaptic proteins associated with an increased risk of dementia progression and showing different magnitudes of change in earlier AD stages compared to later stages using more traditional neuropathological staging. We applied the SuStaIn method using the CSF proteomic data for these proteins and identified two "subtypes" of AD that were determined to fit our data better. These 8 CSF biomarkers showed different progression patterns across the two subtypes. For instance, among these biomarkers, *IGF1* tend to become abnormal in late stages in Subtype1 but predicted to be abnormal in early stages in Subtype2. These observations may suggest that *IGF1* might be a good candidate as an early CSF biomarker for AD. In addition, *NRXN3* (a synaptic cell-cell adhesion molecule) occurs at early stage in Subtype2 but later in Subtype1 which may suggest an earlier increased AD risk for patients in Subtype2 than the ones in Subtype1. In contrast, *PLXNC1* start to become abnormal in early stages in Subtype1 and then became stable before starting showing abnormality again at later stages, whereas in Subtype2 it is only predicted to be abnormal at later stages. These results show that these CSF protein levels identified distinct temporal progression patterns in AD and detected molecular heterogeneity not only among AD cases but also with the presence of control subjects with CDR = 0.

We included these results in the revised manuscript section "Cross-omics integration identified CSF synaptic biomarkers for the molecular staging of AD" (page 28-29, lines 673-692):

"….*Furthermore, we investigated whether these 11 CSF proteins could also identify molecular differences among AD patients and their temporal progression patterns using the Subtype and Stage Inference (SuStaIn) machine learning technique [135,136] (see Methods). Out of the 11 proteins, eight were selected based on QC analyses to be used with SuStaIn. Two subtypes of AD that exhibited distinctive progression patterns based on multiple z-score thresholds (see Methods) were identified (S22A and S22B Fig). While IGF1 tends to become*

abnormal in late stages in Subtype1 (S22C Fig), it is predicted to be abnormal in early stages in Subtype2 (S22D Fig). These observations may suggest that IGF1 might be a good candidate as an early CSF biomarker for AD, as it has been reported previously [130]. In addition, NRXN3 (a synaptic cell-cell adhesion molecule) occurs at an early stage in Subtype2 (S22D Fig) but later in Subtype1 (S22C Fig) which may suggest an earlier increased AD risk for patients in Subtype2 than the ones in Subtype1. In contrast, PLXNC1 started to become abnormal in the early stages in Subtype1 and then became stable before beginning to show abnormality again at later stages (S22C Fig), whereas in Subtype2 it is predicted to be abnormal at later stages (S22D Fig). PLXNC1 was shown to be associated with neuronal loss [137] and therefore, these observations may indicate that PLXNC1 might be a good candidate as a biomarker for monitoring the synapse and neuronal loss. Altogether, these results show that these CSF protein levels identified distinct temporal progression patterns in AD and detected molecular heterogeneity among AD cases and in the presence of control subjects with CDR = 0…."

Disease subvariants and genetic heterogeneity: We would like to mention that we identified similarities and differences in the analytical approaches to the study from Young and collaborators. Alzheimer's Disease has both an Autosomal Dominant (ADAD), and complex genetic architecture. Indeed, carriers of pathogenic mutations in APP, PSEN1, and PSEN2, which we refer to as ADAD, tend to have a distinctive clinical manifestation and a younger age at the onset of cognitive decline compared to sporadic non-carriers, which is referred to as sporadic AD. Our group is highly invested in determining whether the genetic factors identified in sporadic AD play any role in ADAD[1]. We have compared the metabolomic[2], cellular population structure[3], and transcriptional differences at the single cell-type resolution[4] between ADAD and sporadic non-carriers. All our studies indicate that the parietal cortex tends to show a unique and more pronounced aberrant molecular profile than the one we observe in sporadic AD. That is why we did not include different genotypes (ADAD) in this study. Instead, we used unsupervised machine learning approaches to determine whether the brain omics show alternative profiles as emergent properties. We mainly aimed at capturing the heterogeneity in postmortem sporadic AD brains, like the study of FTD heterogeneity among c9orf72 carriers reported by Young *et al.*, 2018 and referenced by the reviewer. We want to mention that we tested whether any of these profiles/clusters are associated with APOE e4 allele, or a polygenic risk score that captures genome-wide risk in sporadic cases. To our surprise and similar to Neff *et al.*, 2021[5], we found no significant association.

These observations were highlighted in several places in the **original** manuscript, including the Results sub-section "A multimodal profile of AD brains is associated with poor cognitive function and molecular attributes" (page 9, line 196):

"…. *The Knight-C4 cluster was not associated with either AD polygenic risk score (S2 Fig), APOE ℰ4 allele carrier status (S3A Fig), or Braak stages (neurofibrillary tangle or amyloid) (S3B and S3C Fig)….*"

We also mentioned these observations in the "Discussion" section (page 28, line 669):

"…. *Similar to previous studies, we did not find a significant association of APOE e4 or PRS with any molecular profile….*".

- Also notable, the study misses relevant references, in particular those that have already presented extended multi-omics integration in AD, proposing both staging and subtyping across multiple populations. The authors should accurately describe the field and compare main results with previous. See for instance:Yasser Iturria-Medina, Quadri Adewale, Ahmed F. Khan, Simon Ducharme, Pedro Rosa-Neto, Kieran O'Donnell, Vladislav A. Petyuk, Serge Gauthier, Philip L De Jager, John Breitner, David A. Bennett, 2022. Unified Epigenomic, Transcriptomic, Proteomic, and Metabolomic Taxonomy of Alzheimer's Disease Progression and Heterogeneity. Science Advances. Vol 8, Issue 46. DOI: 10.1126/sciadv.abo6764

We thank the reviewer for bringing this point to our attention. Although we have included and discussed multiple previous studies addressing pathological and molecular heterogeneity in AD (references 1-9 and 23-32 in the original manuscript) and particularly those that present extended multi-omics integration in AD (Ref. 23-32), we missed the study from Iturria-Medina et al. We have included this study and additional studies in the revised manuscript and extended our "Introduction" and "Discussion" sections to describe previous studies, including this study, in more detail and discuss how our study is different.
The following text was added to the introduction (page 4-5, lines 80-102):

"…Previous studies have found molecular features associated with different AD clinical and pathological profiles [2,3,10]. For instance, CSF proteins for data-driven clustering found an association between hyperplasticity and increased BACE1 levels with clinical differences in AD patients [2]. Similarly, a system biology approach studying transcriptomics from multiple cortical regions identified AD molecular subtypes associated with multiple dysregulated pathways including susceptibility to tau-mediated neurodegeneration, amyloid-β neuroinflammation, synaptic signaling, immune activity, mitochondria organization, and myelination [3]. Despite the substantial effort in these studies to identify molecular associations with clinical and pathological AD features, broader conclusions are limited by its nature, as single-omic analyses typically capture changes in a single component of the biological cascade. Additional studies have leveraged cross-omics data integration approaches to study the alterations of molecular and cellular pathways underlying AD pathophysiology [35,36] providing more evidence that single-omic analyses do not capture most pathways involved in AD pathology. Moreover, CSF multi-omics molecular signatures differentially related to AD pathology have also been identified [24]. Recently, integrating AD brain and blood multi-omics data with clinical and pathological data identified three molecular subtypes and inferred their respective molecular trajectories that diverge from the neuropathologically free control brains [10]. Furthermore, the molecular alterations underlying AD progression and heterogeneity are more clearly understood when studied in multiple brain regions with different burdens of AD pathologies. For instance, the parietal cortex, an understudied brain region affected in later stages of AD [16,37,38], can better capture the more initial molecular changes in AD etiology compared to other severely affected regions (e.g. DLPFC), which will help to identify molecular dysregulations underlying AD progression before a higher burden of Aβ plaque and tangle occurs.…."

We also extended our "Discussion" section to include the following (page 32, lines 769-780):

"….Our results are consistent with previous studies that report molecular correlates with AD clinical data and neuropathological staging [3,10]. We show that AD heterogeneity cannot be

entirely explained by neuropathological variables (e.g. β-amyloid or tau accumulation), APOE ε4 allele carrier status, or even by differences in age or sex. Most of the previous studies have included a single-omic layer [2,3] capturing molecular changes in a single component of the biological cascade, or have analyzed a single AD cohort and a unique brain region (lacking independent replication) [10] as we believe that molecular changes and heterogeneity in AD are better understood when studied in multiple brain regions. Our study leverages multiple AD cohorts (Knight ADRC, MSBB, ROSMAP) and multiple brain regions (parietal cortex, DLPFC, PHG) offering us substantial insights into AD such as our findings of the significant enrichment of AD molecular profiles in synaptic genes and pathways that allow us to identify CSF synaptic biomarkers…..".

- Also, the manuscript should make clear from the introduction the added value in the context of the previous work (no, not all previous AD stratifications have been restricted to a single omic, as claimed in the Introduction). As a suggestion, the authors may accentuate their use of single-nucleus RNA for subtypes-subtypes comparisons.

We agree with the reviewer that this was not described enough in the introduction. We have extended our "Introduction" (page 4-5, lines 80-102) and "Discussion" (page 32, lines 769-780) sections as mentioned in the previous response to describe more the previous work and highlighted the added value of our work in the context of the previous work with the texts shown in the previous response.

- Across the manuscript's text, it is unclear and confusing if the snRNA-seq data was used or not for the subtypes discovery, or only the bulk transcriptomics. Even in the abstract, it reads like if it was considered, which would add value to the analyses in terms of novelty, but on Figure 1 it appears on the other/right side, for post-clustering comparisons. I strongly encourage the authors to make this the clearest possible (it is not clear also in the methods). And, if not considered, to clean all possible insinuations that it was (for example, when claiming in the abstract "to integrate high-throughput bulk and single-nucleus transcriptomic, proteomic, …").

We apologize for the lack of clarity regarding the use of snRNA-seq data in the previous version of the manuscript. Single-nuclei RNA-seq data is still not available for all of the cohorts and was not used in the discovery of cross-omics profiles. Furthermore, the existing single-cell data available is not as comprehensive as transcriptomics data from tissue homogenates (bulk RNA-seq) in terms of subject inclusion or sample size. Therefore, we used bulk RNA-seq data to integrate with other omics to study AD molecular profiles. The single-cell data was used to determine the cell-types expressing those genes that we characteristic in multi-omics studies. We have included more details in this revised version of manuscript and provided more clarifications and description of how the snRNA-seq data was used.
We have updated the sentence in the Abstract (page 2, line 29) and removed single-nucleus word as follows:

"…to integrate high-throughput transcriptomic, proteomic, metabolomic, and lipidomic profiles…".

In addition, we extended the Abstract to explain the uses of snRNA-seq data and elucidate that snRNA-seq data was combined with the identified molecular profiles as follows (page 2, lines 38-39):

"…Furthermore, we leveraged single-nuclei RNA-seq data to identify distinct cell-types that most likely mediate molecular profiles….".

Finally, we updated the title of a results section to reflect this more clearly (page 22, line 511):

"Single-nucleus transcriptomics suggests neurons and alternative glial cells mediate cross-omics profiles of AD"

Reviewer #2:

The study Brain cross-omics integration in Alzheimer's disease by Eteleeb and colleagues is an important study and approach in the analysis and integration of molecular and clinical data in Alzheimer's disease. This topic is now of increasing interest given the significance of the disease, powerful computational methods, and the many available existing and new data sources.

We thank the reviewer for the constructive and supportive feedback. We appreciate the reviewer's recognition of the importance of our work for the field and we extend our gratitude for the supportive comments on the structure of the manuscript.

The introduction is well written and surveys the significance of single cell genomics and AD biomarkers including molecular and trajectory analysis. Cross-omics as proposed in this study approaches reveal how complex biomolecular profiles change in association with a disease and the relationships and correlations between distinct classes of biological molecules. The authors employ a Bayesian integrative clustering method to integrate three omics datasets then applying multiple association analyses and including 144 association with clinical and neuropathological attributes (e.g., sex, age of death, age at onset, post-mortem interval, CDR, Braak amyloid stage, Braak neurofibrillary tangle stage), survival, and differential expression analyses to characterize AD molecular profiles.

We thank the reviewer for this elegant summary of the work and their support.

The figures are in general well done and Figure 1 presents a clear outline of the approach and methodology. The authors discovered four unique multimodal molecular profiles, one showing signs of poor cognitive function, a faster pace of disease progression, shorter survival with the disease, severe neurodegeneration and astrogliosis, and reduced levels of metabolomic profiles. This profile shows similar cellular and molecular profiles in multiple affected cortical regions associated with higher Braak tau scores and significant dysregulation of synapse-related genes and endocytosis, phagosome, signaling pathways altered in AD early and late stages. These are interesting associations illustrating the power of this type of analysis. While the analysis methods presented are largely standard, there are done carefully and statistically valid.

We thank the reviewer for the supportive comments regarding the figures and appreciate their recognition of our work, in particular highlighting the power of this type of analysis.

The capability of cross-omics approaches and its superiority over single-omic analyses as a powerful resource that provides molecular insight into the AD pathogenesis and identifies possible biomarkers for early synaptic dysfunction, cognition, and AD staging. There is opportunity to put forth the the methodology presented as a general approach for data harmonization and analysis in AD/ADRD. There is considerably increased interest in this with several ongoing projects pertaining to data sources such as ROSMAP and the Million Veterans Program from the VA. These and other data source may additionally benefit from the approach here.

We thank the reviewer for raising this point and appreciate their suggestion. We fully agree with the reviewer that there has been an increasing interest in data harmonization and integration. We agree with the reviewer that this approach can be leveraged as a resource for harmonization and analysis in AD/ADRD and we would consider this suggestion for future work.

The networks analysis and pathway analysis including gene ontology results of Figure 9 are I believe less convincing. Many of these association are non very specific with limited p-value significance, and large network presentation such as in Figure 9 are not particularly revealing. The authors may consider tightening the paper with somewhat more focus on key results and the generality of the approach. All in all this is a well done study with interesting findings and potentially a valuable contribution to the analysis and harmonization of AD/ADRD data.

We thank the reviewer for their suggestion. Following their advice, we revamped Figure 9 and placed the Panels D-H from the previous version into a new supplementary figure (S21 Fig). In this way, we believe that the attention of the readers will be focused on the translational aspects of our study, and the importance of synaptic genes as candidates CSF biomarkers for AD and possibly cognition.

New figure 9 (the updated legend is available within the text, page 27, lines 650-657):

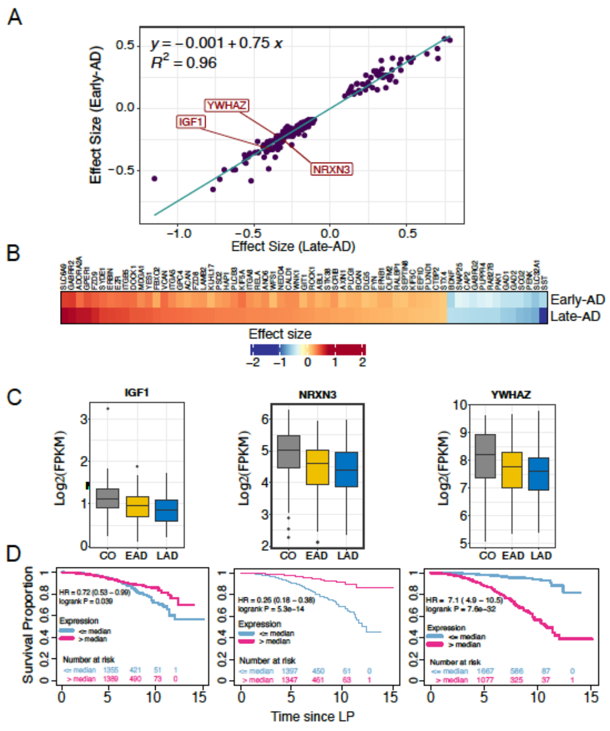

Reviewer #3:

The manuscript "Brain cross-omics integration in Alzheimer's disease" by Eteleeb et al leveraged a large number of omics measurements to identify AD subtypes, followed by their detailed molecular analysis. The topic of AD subtyping has been gaining momentum lately. Perhaps this is afforded because of the accumulation of the omics data. The presented study is on par published on the same topic and is worthy contribution to the overall forum discussing the subtypes of the AD. There are number of issues that I'd like to see addressed prior the publication.

We thank the reviewer for the recognizing the importance of our work and we address their concerns below.

1. Please discuss the choice of the parietal cortex for studying the AD. What was the rationale for selecting this brain region? What are the pros and cons compare to a more conventional one - DLPFC?

We thank the reviewer for bringing this point to our attention. The parietal cortex is an understudied brain region that is affected in later stages of AD[4,6,7]. Thus, at the time of death of most AD patients, the parietal cortex can capture the more initial molecular changes in AD etiology, compared to other severely affected regions that are usually studied in other cohorts, for example the DLPFC for ROSMAP. Furthermore, tissue Microarray data from our collaborator, Dr. Sutherland, Director of the New South Wales – Brain Tissue Resource Center (NSW-BTRC), shows that the parietal cortex has a lower burden of plaques and tangles than the DLPFC, but

higher than other regions, like the inferior temporal cortex. We include this confidential data for evaluation by the reviewers:

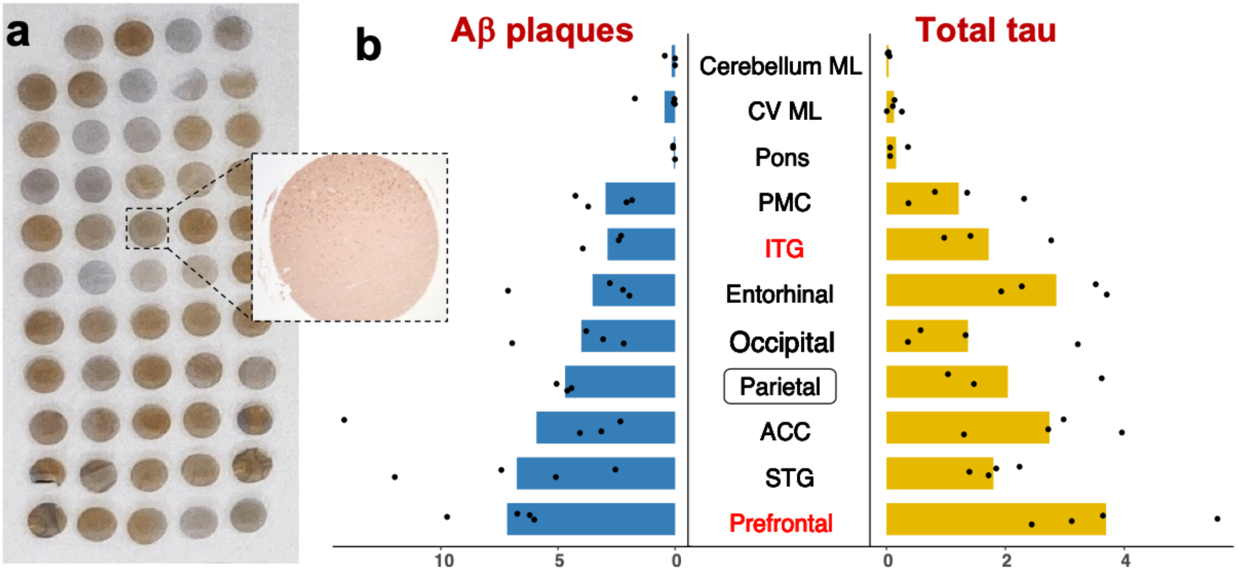

Rebuttal Figure 1 (Confidential): **Neuropathological characterization of global AD pathology in the post-mortem human brain tissue.**

(a) Tissue microarray (TMA) was constructed by drilling 2mm tissue plugs from FFPE blocks of grey and adjacent white matter across the brain and immunostained for Aβ, total tau and NeuN.

(b) Areal fractions of amyloid-plaques (Aβ) and tau/neurofibrillary pathology respectively across the brains of four sporadic AD patients: two males (74 and 80 yo) and two females (78 and 90 yo). Plaques are most dense in the prefrontal cortex (bottom left, highlighted in red color) and lowest in the cerebellum. Neurofibrillary pathology was most dense in prefrontal cortex (bottom right, highlighted in red color) and lowest in the cerebellum. The parietal cortex presents intermediate Aβ and neurofibrillary pathology.

Thus, we used data from parietal cortex in our discovery stage, to study the aberrant and dysregulated molecules in the presence of AD pathological lesions, before a higher burden of Aβ plaques and NFTs occurs along extended periods of times, and possibly additional compensatory effects confound initial changes associated with etiology. We replicated our initial findings in two more brain regions, the DLPFC in ROSMAP cohort and parahippocampal gyrus (BA36) from the MSBB cohort. We believe that studying multiple regions with different burdens of Aβ and plaques will allow us to better understand the molecular changes associated with the progression of the disease, but at the present time, we are constrained to generate more data systematically in multiple brain regions from the same donors.

Since we believe this is an important point that should be included in the revised manuscript, we have extended our "Discussion" section to include the above illustration of the choice of using this region (page 29, lines 699-705):

"*… In the discovery stage, we used data from the parietal cortex, an understudied brain region affected in later stages of AD [16,37,38]. The parietal cortex was chosen to study*

dysregulated molecules in the presence of AD pathological lesions before a higher burden of Aβ plaques and NFTs occurs along extended periods of time, and possibly additional compensatory effects confound initial changes associated with etiology. In addition, the parietal cortex typically captures the more initial molecular changes in AD etiology compared to other severely affected regions (e.g., DLPFC), making it a suitable region for our study…."

2. The main finding of the manuscript are the 4 clusters or subtypes of AD. Since the code was not available, I could only review the analysis based on the description in the methods section. Use of BIC and deviance criteria for selection of the number of clusters is a good choice. However, one discrepancy needs a little bit more effort to explain. The supplementary figure 1 states that the optimum is at number of clusters 3 (if I am reading the X axis right). The legend to the same figure states that K=3, thus the samples are divided into 4 clusters. This is confusing. Please double check the number of clusters and clarify the rationale and the arithmetic in the figure and the legend.

We thank the reviewer for bringing this point to our attention and we apologize for the confusion on details on selecting the number of clusters. iClusterBayes[8] fits multiple clustering solutions based on a number of clusters (k, aka eigne features) and then uses Bayesian information criteria (BIC) and deviance ratio to select the optimal model. When BIC value reaches the minimum and the deviance ratio reaches a plateau at a specific K value, the optimal clustering solution with that K value is selected which indicates that the model fits the data best when the samples are divided into K + 1 clusters and therefore the number of clusters will be *k* + 1. In the previous version of the manuscript, we chose to follow the nomenclature used by the developers of the method, and this critical information was provided in Supplementary Figure 1 legend. However, to avoid any confusion and enhance the readability of the manuscript, we have updated Supplementary Figure 1 and its legend as shown below:

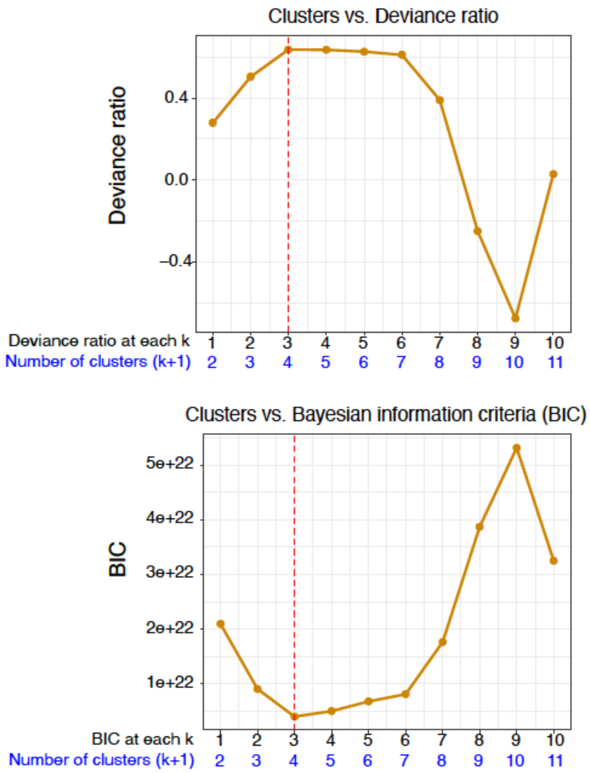

Rebuttal Figure 2. The deviance ratio (top) and the Bayesian information criterion—BIC (bottom) at each K (number of eigen features). The optimal solution is observed with K =3 when the BIC is the minimum and the deviance ratio is the maximum dividing our samples into four clusters (k+1).

We have uploaded our analysis code used to integrate all cross-omics data for the three cohorts as well as all downstream analyses to our GitHub repository which can be found at https://github.com/HarariLab/Cross-omics-Integration.

3. The discussion section is completely missing other studies that investigated the number of subtypes of AD. Please discuss your results within the context of other findings.

We thank the reviewer for this suggestion. We recognize that we had included multiple previous studies addressing pathological and molecular subtypes in AD in the introduction section in the previous version of the manuscript (references 1-9), and now provided a more comprehensive description in this new version. We recognize that our findings should also be contextualized in the discussion. Thus, we have extended our "Discussion" in the revised manuscript to contextualize our results within the previous studies that investigate AD molecular heterogeneity using multi-omics data.

The following paragraph was added to the revised manuscript (page 32, lines 769-780):

"….Our results are consistent with previous studies that report molecular correlates with AD clinical data and neuropathological staging [3,10]. We show that AD heterogeneity cannot be entirely explained by neuropathological variables (e.g. β-amyloid or tau accumulation), APOE ℰ4

allele carrier status, or even by differences in age or sex. Most of the previous studies have included a single-omic layer [2,3] capturing molecular changes in a single component of the biological cascade, or have analyzed a single AD cohort and a unique brain region (lacking independent replication) [10] as we believe that molecular changes and heterogeneity in AD are better understood when studied in multiple brain regions. Our study leverages multiple AD cohorts (Knight ADRC, MSBB, ROSMAP) and multiple brain regions (parietal cortex, DLPFC, PHG) offering us substantial insights into AD such as our findings of the significant enrichment of AD molecular profiles in synaptic genes and pathways that allow us to identify CSF synaptic biomarkers….".

4. Legend of Figure 1 (page 6, line 128) states "genes=60,754". Human genome is limited to 20K or so genes. It isn't clear what authors referring to. Are these collective measurements of the canonical mRNA transcripts across 3 cohorts or number of transcripts including splice isoforms? I'll leave this up to authors discretion if they should switch to the term mRNA transcript rather than gene.

We thank the reviewer for highlighting this point and we apologize for the lack of clarity. The 60,754 represents all features in the transcriptome including protein-coding genes, long noncoding RNAs, small RNAs, pseudogene, etc. It is a comprehensive gene annotation data. We have clarified this in the revised manuscript and used the term "features" to represent all entries/bio-features in the entire transcriptome.

5. The term "cross-omics" triggers a minor question. Did the authors decide to use the term "cross-omic" to set themselves apart from the rest of the studies that use the term "multi-omic"? Moreover, once sentence in the discussion section uses both terms in one sentence (page 28, line 664). If there is no good rationale for using a distinct term, please consider switching to "multi-omic". Though I leave this issue to author's discretion too.

We thank the reviewer for raising this interesting point. We fully agree with the reviewer that there is no a clearly established difference between multi-omics and cross-omics yet. Indeed, these terms are sometimes interchanged.

It is our understanding that multi-omics approaches focus on the simultaneous profiling of multi-omics or other modalities from the same cells[9,10]. In fact, this term is currently being used to describe platforms and chemistries that allow the generation of gene expression and ATAC-seq in parallel in spatial resolved transcriptomics and similarly 10X Genomics multiome kits.

Nowadays there is a tendency to refer as cross-omics to those approaches that integrate and analyze multiple omics data sets from different bio-samples. Indeed, this term has been employed in multiple other manuscripts and areas[11], including T2D[12], Parkinson Disease[13], aggregation of summary data[14] and White Matter hyperintensities[15].

We are adopting this new term, as we believe it reflects more accurately our work.

6. Please state how exactly code will be shared. I really hope it won't be "available upon request". Though ideally, I'd prefer the code available at the time of the review.

We thank the reviewer for the interest in looking at our code. We originally stated that the analysis code used for this study will be available at the time of publication, but in responding to the reviewer's request, the analysis code used to integrate cross-omics data for the three cohorts as well as all downstream analyses is now available at https://github.com/HarariLab/Cross-omics-Integration

References

1. Cruchaga C, Del-Aguila JL, Saef B, et al. Polygenic risk score of sporadic late-onset Alzheimer's disease reveals a shared architecture with the familial and early-onset forms. *Alzheimer's & Dementia*. 2018;14(2):205-214.
2. Novotny BC, Fernandez MV, Wang C, et al. Metabolomic and lipidomic signatures in autosomal dominant and late-onset Alzheimer's disease brains. *Alzheimers Dement*. Published online October 17, 2022. doi:10.1002/alz.12800
3. Li Z, Del-Aguila JL, Dube U, et al. Genetic variants associated with Alzheimer's disease confer different cerebral cortex cell-type population structure. *Genome medicine*. 2018;10(1):1-19.
4. Brase L, You SF, D'Oliveira Albanus R, et al. Single-nucleus RNA-sequencing of autosomal dominant Alzheimer disease and risk variant carriers. *Nature communications*. 2023;14(1):2314.
5. Neff RA, Wang M, Vatansever S, et al. Molecular subtyping of Alzheimer's disease using RNA sequencing data reveals novel mechanisms and targets. *Science advances*. 2021;7(2):eabb5398.
6. Elahi FM, Miller BL. A clinicopathological approach to the diagnosis of dementia. *Nature Reviews Neurology*. 2017;13(8):457-476.
7. Smith AD. Imaging the progression of Alzheimer pathology through the brain. *Proc Natl Acad Sci USA*. 2002;99(7):4135-4137. doi:10.1073/pnas.082107399
8. Mo Q, Shen R, Guo C, Vannucci M, Chan KS, Hilsenbeck SG. A fully Bayesian latent variable model for integrative clustering analysis of multi-type omics data. *Biostatistics*. 2018;19(1):71-86.
9. Bredikhin D, Kats I, Stegle O. MUON: multimodal omics analysis framework. *Genome Biol*. 2022;23(1):42. doi:10.1186/s13059-021-02577-8
10. Augustijn D, de Groot HJ, Alia A. HR-MAS NMR applications in plant metabolomics. *Molecules*. 2021;26(4):931.
11. Kerkhofs MH, Haijes HA, Willemsen A, et al. Cross-omics: integrating genomics with metabolomics in clinical diagnostics. *Metabolites*. 2020;10(5):206.
12. Liu J, Carnero-Montoro E, van Dongen J, et al. An integrative cross-omics analysis of DNA methylation sites of glucose and insulin homeostasis. *Nature communications*. 2019;10(1):2581.
13. McIntyre LM, Huertas F, Morse AM, et al. GAIT-GM integrative cross-omics analyses reveal cholinergic defects in a C. elegans model of Parkinson's disease. *Scientific reports*. 2022;12(1):3268.
14. Chen L, Lu Y, Oliva M, Pierce B, Liu J. Integrative cross-omics and cross-context analysis elucidates molecular links underlying genetic effects on complex traits. Published online 2022. Accessed December 21, 2023. https://www.researchsquare.com/article/rs-2137179/latest
15. Yang Y, Knol MJ, Wang R, et al. Epigenetic and integrative cross-omics analyses of cerebral white matter hyperintensities on MRI. *Brain*. 2023;146(2):492-506.
