# Supplementary material for: Brain high-throughput multi-omics data reveal molecular heterogeneity in Alzheimer’s disease
Source: PLoS Biol. 2024 Apr 30;22(4):e3002607. doi: 10.1371/journal.pbio.3002607 (PMC11086901; doi:10.1371/journal.pbio.3002607)
Supplement: Attachment — Submitted filename: Rebuttal_Letter_PLoSBio_Resub2_Final.pdf [file pbio.3002607.s039.pdf]

Reviewer's Comments

We thank the editors and reviewers for evaluating our manuscript again and for their valuable comments and supportive feedback. We are excited that reviewers 2 and 3 found our revised version suitable for publication. We appreciate all reviewers' help in improving the final version of our manuscript. Despite the substantial effort we have made to address critiques raised by reviewer 1, they still have these concerns that we must respectfully disagree. Below, we addressed their comments point-by-point. Changes to the manuscript have been highlighted in a 'track-changes' version.

Reviewer #1:

I believe that while the study involve an impressive amount of data, it still suffers from major pitfalls, starting at the methodological level, and that many of the authors' replies don't address effectively the raised points:
- Independently of if detecting subtypes or profiles or variants, the use of clustering directly on data from an heterogenous population is fundamentally wrong. Although the participants were neuropathologically confirmed as AD, they surely were at different points on the disease's long timeline (and having different level of neuropathological and clinical severity, plus of course different levels of molecular alterations just due to progression differences), and just throwing the data into clustering would actually imply a lot of confounding effects in the discovery analysis.

We thank the reviewer for noticing and highlighting "the impressive amount of data". Our study is intended to use data-driven approach to identify data emergent molecular profiles of AD, characterize them, and identify their clinical and biologically relevance.
We respectfully disagree with the reviewer. In the real world, human brain tissue from AD patients and controls are heterogeneous. Neuropathological data collection standards have changed, and our understanding of the disease has progressed. As brains are usually characterized soon after death, and seldom cases neuropathological reports are retrospectively done, the level of detail and completeness of data is condition to time of autopsy, that is variable in the case of large cohorts of human brains collected through efforts spamming many years. Thus, our approach was to analyze the data without stratification by any neuropathological or clinical variables. In addition, this can introduce artifacts, as these provide a discreet categorization instead of continuous values (quantitative neuropathological variables) as expected with disease progression. Instead of using the Braak stages or CDR as endpoints, we chose a data-driven characterization of the molecular heterogeneity. By virtue of this approach, we identified and replicated molecular profiles in multiple cohorts and brain regions that are robust and biologically meaningful.

- We will always find clusters, or profiles, but these can be deeply affected or influenced by how aligned where the subjects at the time of analysis. If not at same or close stage, the clusters although statistically 'valid', will be biologically meaningless.

We respectfully disagree with the reviewer again. As we mentioned in the previous response, our work is intended to use a data-driven approach to identify robust molecular signatures that can be validated across multiple cohorts and brain regions. We reiterate that all human brains were collected postmortem, and their disease progression stage cannot be controlled, as in experimental models. Instead, we employed an unsupervised approach in which we initially clustered molecular profiles and then identified the associated neuropathological or clinical variables. This unsupervised approach is often used in high-throughput *omic* and system biology approaches. We respect the reviewer's point of view on the importance of co-variates and how they can affect molecular profiles. We included all those variables in our statistical models, and yet molecular signatures emerged from our unsupervised analyses, proving the hypothesis in a discovery dataset. Our replication efforts in different cohorts and the validation using CSF levels of some of the markers from the molecular signatures discovered in brain provide biological relevance to our results. It is out of the scope of the current manuscript but future studies could validate our findings using model organisms at different stages of the disease in more controlled and homogenous conditions.

- Once the clusters are detected from a mixed population (in terms of progression), all the subsequent analysis are affected by it, and unfortunately all discoveries will be strongly biased.

We apologize, but we disagree with the reviewer here as well, respectfully. In this work, we are not aiming to detect disease progression, and our robust findings prove that even using an unsupervised multi-omics approach can find common underlying signatures. Indeed, in our experience, the human tissue from the multiple brain banks we analyzed does not provide sufficient/balanced data points in different disease stages (e.g., Braak stages) to perform a systematic study of molecular changes associated with disease progression.

- BTW, the need to use Sustain on a very significantly small subset of markers is unjustified and somehow disconnected from the rest.

We used this approach to further characterize AD molecular profiles by using a set of the specific CSF biomarkers we identified in our study of brain molecular data. By selecting these, we can better characterize these profiles. A large untargeted study of CSF biomarkers is beyond the scope of this study.

- The use of data from multiple brain regions is claimed as a novelty of the manuscript, however, in my opinion the authors don't really show the advantages or disadvantages of it. It is explicitly said that it is better, but it is not studied, and not shown with clarity.

We respectfully disagree with the reviewer. In this study, we leveraged multiple brain regions from multiple cohorts in a discovery and replication approach. Molecular profiles of AD were discovered in one cohort (Knight ADRC) and specific cortical region (parietal cortex) and replicated in two additional cohorts (MSBB, ROSMAP) that ascertained two additional regions (PHG, DLPFC). In this study, we have shown that distinct molecular profiles of AD are present across brain regions.

Again, clusters (or profiles) and subsequent significant differences in post-hoc analyses will always appear. My impression is that, sadly, the authors try to reply/convince by points (more data, more analyses, more figures, etc.), but there is still an underlying lack of understanding on the meaning of biological profiles (or clusters or subtypes or subgroups, name is irrelevant) to study disease heterogeneity.

We respectfully disagree with the reviewer. In our experience, the replication of emergent data-driven properties is a much sought-after result from high-throughput molecular studies. Identifying these clusters and molecular profiles in multiple cortical regions and cohorts provides strong evidence of the biology captured in this study. The reviewer seems to understand biological profiles are non-overlapping, finite, and defined units like categories (Braak stages) across disease staging. We are implementing a novel and unbiased data-driven approach across the spectrum of disease changes in the post-mortem brain tissue to identify data emergent molecular (biological) profiles with biological or biomedical relevance.

To summarize the points raised by the reviewer, we provided additional details and discuss these points as limitations in the Discussion section (page 32-33, line 787 and line 799) as follows:

"…We showed that distinct molecular profiles of AD are present in multiple brain regions by leveraging multiple AD cohorts (Knight ADRC, MSBB, ROSMAP) and multiple brain regions (parietal cortex, DLPFC, PHG)…"

"…Large number of brain tissue, possibly from donors at multiple Braak staging, including better representation of those initial stages, coupled with quantitative neuropath will help elucidate further AD heterogeneity…."

Reviewer #2:

The authors have made a large effort to meaningfully address my critiques and the critiques of other reviewers, I believe, and the paper is much improved

We thank the reviewer for the supportive feedback and appreciate their recognition of our effort to address their critiques and improve our manuscript.

Reviewer #3:

The authors addressed all of my raised concerns and improved the manuscript.

We thank the reviewer for recognizing our effort to improve the manuscript and we are happy that were able to address their concerns.
